# Sexual and reproductive health (SRH) needs for forcibly displaced adolescent girls and young women (10–24 years old) in humanitarian settings: a mixed-methods systematic review

**DOI:** 10.1186/s12978-023-01715-8

**Published:** 2023-11-24

**Authors:** Rachel E. Soeiro, José Paulo de Siqueira Guida, Juliana da-Costa-Santos, Maria Laura Costa

**Affiliations:** Department of Obstetrics and Gynecology, Universidade Estadual de Campinas (UNICAMP), 101 Alexander Fleming St, Campinas, SP Brazil

**Keywords:** Sexual and reproductive health, Adolescent, Young women, Knowledge, Refugee, Migrant, Systematic review

## Abstract

**Background:**

Globally, there are 42 million women and girls estimated to be forcibly displaced. Adolescent girls and young women in humanitarian settings have their sexual and reproductive health (SRH) neglected. This systematic review aimed to describe SRH obstacles that adolescent girls and young women (10–24 years old) face in humanitarian settings in line with the Sustainable Development Goals.

**Methods:**

We conducted a mixed-methods systematic review in six databases, focusing on migrant women ages 10 – 24and their SRH outcomes. The mixed-methods appraisal tool was used to evaluate the quality of the studies. This review follows PRISMA and the Systematic Review Guidelines from the Centre for Reviews and Dissemination recommendations.

**Results:**

Among the 1290 studies screened by abstracts, 32 met the eligibility criteria: 15 were qualitative, 10 were quantitative and seven were mixed-methods studies. Most studies were performed in the last four years, in African countries. They discussed the increased frequency of adolescent pregnancies (16–23%), lack of contraceptive use and access (8–32%), poor menstrual hygiene management (lack of water, shortage of menstrual hygiene supplies), ignorance and stigma about sexually transmitted infections and HIV, a higher number of child, early and forced marriage or partnership and sexual and gender-based violence, challenging to obtain SRH information/knowledge/access, and unmet SRH needs.

**Conclusion:**

Migration is a current issue. Although there is a growing number of studies on adolescent girls and young women’s SRH in humanitarian settings, this population remains overlooked, and face several challenges in SRH. There is a need for targeting interventions on SRH.

**Supplementary Information:**

The online version contains supplementary material available at 10.1186/s12978-023-01715-8.

## Plain language summary

This systematic review describes the available evidence on the sexual and reproductive health (SRH) challenges faced by adolescent girls and young women in humanitarian settings. Thirty-two studies were analyzed, demonstrating poor SRH outcomes: higher incidence of adolescent pregnancy, lack of access to contraceptives and low rates of its use, precarious menstrual hygiene management (MHM), ignorance and stigma about sexually transmitted infections (STIs) and HIV, higher rates of child, early and forced marriage or partnership (CEFMP) and sexual and gender-based violence (SGBV). Identifying these challenges can help humanitarian actors address the SRH needs of these populations.

**Supplementary Information:**

The online version contains supplementary material available at 10.1186/s12978-023-01715-8.

## Background

In 2022, the United Nations High Commissioner for Refugees (UNHCR) estimated that 100 million people were forcibly displaced worldwide due to conflict, violence, and weather-related events such as floods, storms, and cyclones [1]. The Internal Displacement Monitoring Center (IDMC) reported that nearly 42 million of the displaced people were women and girls, 65% from African and Middle Eastern countries. [2].

The Sustainable Development Goals (SDGs) aim to reduce maternal mortality (Goal 3.1), ensure universal access to sexual and reproductive health services (Goal 3.7), end all forms of violence against all women and girls (Goal 5.2), and end child marriage (Goal 5.3) by 2030 [3]. These targets are interlinked and have an impact in the sexual and reproductive health (SRH).

Despite of the SDGs, sexual and reproductive health needs for migrant adolescent girls and young women (10–24 years old) in humanitarian settings remain unmet [4].Studies have described the lack of access and inequalities regarding SRH for migrant adolescent girls and young women (AGYW), including language barriers, difficulties in obtaining contraceptives, fees, waiting times, travel distances, and the insufficiency of specific programs for this population [4–8]. They have higher rates of repeated and unsafe abortions, lower antenatal care (ANC) attendance, more postpartum complications such as perinatal mortality, fetal death and stillbirth, and a higher risk of HIV and sexual violence [7–9]. Approximately 60% of maternal deaths or childbirth among adolescent girls occur in conflict or disaster contexts [9].

The discussion of SRH needs for AGYW in humanitarian settings has gained global attention in recent years, however there are gaps in the collection and systematization of comprehensive data, making their use in policy design and implementation in these scenarios challenging and further away from the SDGs [10].

This systematic review aims to explore the current qualitative and quantitative research landscape on SRH issues of adolescent girls and young women displaced by humanitarian crises living in fragile settings in line with the SDGs, given the amount of recent new studies.

## Methods

### Search strategy and study design

This mixed-methods systematic review was conducted according to Sandelowski et al. [11] and followed the three steps: segregated (qualitative and quantitative studies were analyzed separately), integrated (the differences between qualitative and quantitative studies were minimized), and contingent (addressing the same research questions).

We followed the reporting guidelines described in the Preferred Reporting Items for Systematic Reviews and Meta-Analyses (PRISMA) statement and the Systematic Review Guidelines from the Centre for Reviews and Dissemination [12, 13]. For the studies’ evaluation, we used the “Additional file 1: Mixed Methods Appraisal Tool (MMAT) version 2018” [14].

The MMAT includes research evaluative criteria for quantitative, qualitative, and mixed-method studies. This tool was first published in 2009 and revised and upgraded in 2018 [15–17]. A user manual with an algorithm guides the studies' analyses. [15].

This review is registered in the PROSPERO platform under the registration number CRD42023403907.

Our research question was "*What is the available evidence on sexual and reproductive health among migrant girls and young women in humanitarian settings?*"

The search strategy (Additional file 1: Annex S1) was built with the guidance of an information specialist. The chosen research terms and their variations, including Medical Subject Headings (MeSH)[18], were combined according to each database's requirements and specifications.

The databases used for searching were PUBMED, PUBMED PMC, EMBASE, BVS / LILACS, SCOPUS, and WEB OF SCIENCE. The syntax was: "adolescent", "reproductive health", and "refugees"; related words such as plurals and alternative forms of the terms (e.g., youth) were also included. A detailed search strategy is available in Additional file 1: Annex S1.

### Eligibility criteria

Original quantitative and qualitative studies which investigated sexual and reproductive health outcomes from the perspective of migrant (defined as displaced women such as refugees, asylum seekers or internally displaced people) adolescent girls and young women (10–24 years old), published until January 05, 2023 (with no lower range), written in English, Spanish or French, were included.

### Exclusion criteria

Editorials, opinion articles, letter to editors, call for action, short reports, brief communications, protocol guidelines, book chapters, retrospective studies, congress annals, newsletters, and other reviews were excluded. The research team opted not to consider grey literature.

**Fig. 1:**
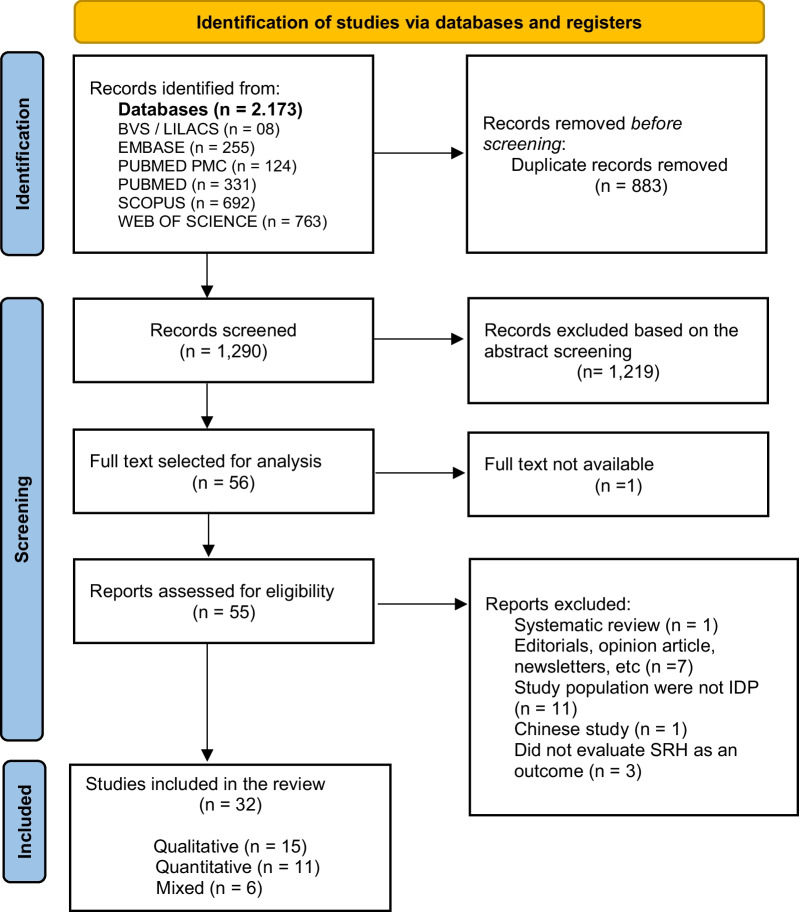
PRISMA 2020 flow diagram: process of the integrative review

Figure 1 shows the process of the review according to the PRISMA guidelines [13].

### Critical appraisal

The search fields were title and abstract. The Rayyan Systematic Review Tool [19] was initially used to screen abstracts and titles.

Two independent reviewers performed the initial blind screening and selection of studies, considering the title and abstract. The study coordinator approached conflicts of selection and made the final decision of inclusion or exclusion among conflicting evaluations.

The full texts were read assessing quality and appropriateness.

To evaluate the quality of the included studies, the “Mixed Methods Appraisal Tool (MMAT) version 2018” [17] was used. The MMAT user manual instructs to provide the analysis with a detailed presentation of each one of the five core criteria, according to the study category [15].

The latest MMAT version, 2018, (Additional file 2: Annex S2) includes two screening questions and a total of 25 criteria, divided into five methodological categories of study designs: (a) qualitative, (b) randomized controlled trials, (c) nonrandomized, (d) quantitative descriptive studies and (e) mixed methods. For each category, there are five core criteria rated in three response options: "yes" (the criterion is met), "no" (the criterion is not met) or "can’t tell" (there is not enough information in the paper to evaluate if the criterion is met or not). Mixed-method studies must be assessed in both, qualitative and quantitative categories and then reevaluated into the integration of quantitative and qualitative studies. [15, 17].

### Data extraction and analysis

Data from all included articles were extracted following the headlines: authors and title, year, study setting, sample size, study population, age of participants, main objectives, study design and conclusions/recommendations (Table 1). Each study was evaluated under its methodological categories. As each category has five items, we considered 20% for each, scoring 100%. Mixed-methods studies were evaluated as mixed-method, and qualitative and quantitative components were independently assessed on their corresponding quality criteria. All articles were given a final quality score, all articles analysed were included in the study (Table 2).

**Table 1:** Study characteristics

	Author/Title	Year	Study setting	Sample size	Study population	Age of participants	Main objectives	Study design	Conclusions/recommendations
1	Asnong et al. Adolescents’ perceptions and experiences of pregnancy in refugee and migrant communities on the Thailand-Myanmar border: a qualitative study	2018	Two refugee camps (Thailand/ Myanmar border)	Total sample: 40 participants (20 pregnant adolescents, 3 husbands of pregnant adolescents, 6 adolescent boys, 6 non-pregnant adolescent girls, 5 locally trained ANC staff members)	Migrants and refugee pregnant adolescents’ girls attending ANC and non pregnant adolescents’ girls	13–19	Explore adolescent pregnancy including the experience, consequences, family, and community support, and SRH knowledge on two refugee camps	Qualitative Cross-sectional FGDs and individual interviews	Adolescents refer to pregnancy as a challenging life event It is necessary to provide comprehensive adolescent friendly SRH services and education to the refugee and migrant communities on the Thailand-Myanmar border
2	Bakesiima et al. Modern contraceptive use among female refugee adolescents in northern Uganda: prevalence and associated factors	2020	Palabek refugee settlement (Northern Uganda)	839 adolescent girls	Sexually active female refugees	15–19	Establish the prevalence and associated factors with modern contraceptive use among female refugee adolescents in Uganda	Quantitative Cross-sectional Questionnaire	Modern contraceptive use was less than 10% among refugee adolescents, putting them at pregnancy risk and its consequences during adolescence. The main reasons for not using modern contraceptives were partner prohibition, fear of side effects, and lack of knowledge This evidence an urgent need for access to quality SRH services and for SRH counselling to empower adolescent girls in refugee settings
3	Benner et al. Reproductive health and quality of life of young Burmese refugees in Thailand	2010	Two refugee camps (Thailand)	416 (222 adolescent boys and 194 adolescent girls)	Young Burmese refugee's	15—24 years	Assess young Burmese refugee's reproductive health issues and quality of life	Quantitative and Qualitative Cross-sectional Self-responded questionnaire Semi-structured questionnaires	There is a lack of sexual health information and SRH services. There is a need for specific policies
4	Bol et al. Pregnancy among adolescent girls in humanitarian settings: a case in refugee camp of Gambella regional state, community-based cross-sectional study, Southwest Ethiopia, 2021	2021	Nguenyyiel Refugee Camp (Gambella region, Southwest Ethiopia)	414 (adolescent girls)	Adolescent girls living in the Nguenyyiel Refugee Camp for at least 6 months before the data collection	10 –19	Determine the prevalence of pregnancy among adolescent girls and associated factors in a Refugee Camp, in the Southwest of Ethiopia	Quantitative Cross-sectional Questionnaire HCG pregnancy test	There is a high prevalence of pregnancy among refugee adolescent girls in the Nguenyyiel Refugee Camp, most among late-stage, illiterate, and those adolescents living without a biological family It is necessary to provide comprehensive adolescent-friendly SRH services and education Future researchers should address other important points such as unmet family planning needs and unwanted pregnancy and use a mixed approach (qualitative and quantitative study)
5	El Ayoubi et al. Sexual and Reproductive Health Information and Experiences Among Syrian Refugee Adolescent Girls in Lebanon	2021	Lebanon’s Bekaa governorate	There is no description of the total number of participants: 3 FG with unmarried adolescent girls (5–7 participants/FG), 11 IDIs with married adolescent girls, and 2 FG with mothers of 11- to 14-year-old adolescent girls (4–8 participants/ FG)	Married and unmarried Syrian refugee adolescent girls	14–20	Understand what SRH information Syrian refugee adolescent girls receive and from which sources	Qualitative Cross-sectional FGDs and individual in-depth interviews (IDI)	There are misconceptions regarding SRH information. The preferred source of information of the married and unmarried adolescent girls was their mothers, followed by schools` and NGO`s SRH sessions, and their peers The SRH programs for refugee adolescent girls should engage their mothers improving their SRH knowledge and communication skills
6	Elnakib et al. Drivers and consequences of child marriage in a context of protracted displacement: a qualitative study among Syrian refugees in Egypt	2021	Giza, Damietta, and Qalyubia (Egypt)	72 (27 married and unmarried adolescent girls, 14 adolescent girls’ mothers, 3 adolescent girls’ fathers, 9 Community Leaders, 6 Health Providers, 11 Humanitarians, 2 Legal experts)	Married and unmarried Syrian refugee adolescent girls	10–19	Understand the drivers of child marriage in a displacement context and how this affects the Syrian refugees girl's wellbeing	Qualitative Cross-sectional FGDs and individual in-depth interviews (IDI)	The study provides an overview of the marriage of adolescent Syrian refugee girls living in Egypt. The interventions should focus not only on the prevention of child marriage but also on mitigating their impacts
7	Ganle et al. Risky sexual behavior and contraceptive use in contexts of displacement: insights from a cross-sectional survey of female adolescent refugees in Ghana	2019	Budumburam refugee camp (Ghana)	242 (adolescent girls)	Refugee adolescent girls	14–19	Assess contraceptive use and sexual behavior among female adolescent refugees in Ghana	Quantitative Cross-sectional Structured questionnaire	The use of modern contraceptives, their knowledge and access to them among refugee adolescent girls are very low
8	Ghandour et al. Coming of age: a qualitative study of adolescent girls’ menstrual preparedness in Palestinian refugee camps in the West Bank and Jordan	2022	Palestinian refugee camps in the West Bank and Jordan	232 (adolescent girls)	Palestinian refugee adolescent girls	14–21	Understand how Palestinian adolescent girls were prepared for menstruation and the main factors influencing their preparedness	Qualitative Cross-sectional FGDs and individual in-depth interviews (IDI)	There is a need for evidence-based interventions regarding SRH (of refugee adolescent girls to address these disparities
9	Goers et al. Child marriage among displaced populations—a 2019 study in Kurdistan Region of Iraq	2022	Governorates of Sulaimani, Erbil, Dohuk (Kurdistan Region of Iraq)	3040 (AGYW)	Host, internally displaced (IDP) and refugee adolescent girls from KRI	10–24	Describe child marriage prevalence, influences, and beliefs among displaced populations in the Kurdistan Region of Iraq (KRI)	Quantitative Cross-sectional Survey	IDP adolescent girls have an increased risk for child marriage than refugee and host adolescent girls in KRI
10	Ivanova et al. A cross-sectional mixed-methods study of sexual and reproductive health knowledge, experiences, and access to services among refugee adolescent girls in the Nakivale refugee settlement, Uganda	2019	Nakivale refugee settlement (Isingiro District, Southwest Uganda)	260 (adolescent girls)	Refugee adolescent girls	13–19	Provide an overview of SRH experiences, knowledge, and access to services among adolescent refugee girls in a humanitarian setting in Uganda	Quantitative and Qualitative Cross-sectional Questionnaire Semi-structured questionnaires and individual in-depth interviews (IDI)	Refugee adolescent girls in humanitarian settings have limited access to SRH services and SRH knowledge. The schools and the parents are their main sources of SRH information A multi-sectoral approach is needed for SRH education and access to SRH services for adolescents. It is also important to offer comprehensive care to sexual violence victims
11	Kågesten et al. Transitions into puberty and access to sexual and reproductive health information in two humanitarian settings: cross-sectional survey of very young adolescents from Somalia and Myanmar	2017	Thailand-Myanmar border and Kobe refugee camp (Ethiopia)	406 Somali VYA girls 399 VYA girls from Myanmar	Refugee adolescent girls from Somalia and from Myanmar	10–14	Describes the characteristics of very young adolescents (VYA) in two humanitarian settings, regarding transitions into puberty, MHM, and access to SRH information	Quantitative Cross-sectional Structured questionnaire	There is a lack of SRH information and supplies for MHM in these two humanitarian settings. VYA's parents are their main source of SRH information SRH interventions involving parents and educational centers may have encouraging results on the VYA pubertal and sexual development
12	Kemigisha et al. A Qualitative Study Exploring Menstruation Experiences and Practices among Adolescent Girls Living in the Nakivale Refugee Settlement, Uganda	2020	Nakivale refugee settlement (Isingiro District, Southwest Uganda)	28 (adolescent girls)	Refugee adolescent girls	13–19	Describe the context and challenges faced by adolescent refugee girls during migration and their stay at the refugee settlement in Uganda and address the knowledge gap	Qualitative Cross-sectional FGDs and interviews	Refugee adolescent girls in humanitarian settings have poor menstrual health management (MHM). It is mandatory to provide timely and evidence-based information. Family and school have an important role in this process
13	Knox How they see it: young women's views on early marriage in a post-conflict setting	2017	Nahr el Bared Palestinian refugee camp (North Lebanon)	37 adolescent girls, 5 adolescent girls’ mothers and 12 NGO workers	Refugee girls from and residing in Nahrel Bared, married engaged to Palestinian refugee men from and residing in Nahr el Bared		Explore the views and experiences of early marriage among married and engaged refugee adolescent girls	Qualitative Cross-sectional FGDs and individual in-depth interviews (IDI)	The refugee adolescent girls residing in Nahr el Bared do not feel forced to marry and did not see themselves as victims. Early marriage was related to insecurity, isolation and loss of friendships and peers. There is a lack of information and misinformation regarding SRH. Any intervention in early marriage must include the community and bring adolescent girls together providing access to courses, leadership, and civic engagement
14	Korri et al. Sexual and reproductive health of Syrian refugee adolescent girls: a qualitative study using focus group discussions in an urban setting in Lebanon	2021	Bourj Hammoud (Urban setting in Lebanon)	40 (adolescent girls)	Married and unmarried Kurdish and Arab Syrian refugee adolescent girls	13–17	Explore the SRH perceptions and experiences of Syrian refugee adolescent girls living in an urban setting in Lebanon	Qualitative Cross-sectional FGDs	There is a need for solid information about SRH issues, through accessible programs adolescents and additionally, encouraging the role of mothers perceived as trusted and accessible sources of information
15	Lee et al. Sexual and reproductive health needs and risks of very young adolescent refugees and migrants from Myanmar living in Thailand	2017	Mae Sot (Myanmar-Thailand border) and Mae La Refugee camp (Thailand)	180 participants (88 adolescent boys, 88 adolescent girls, 4 adolescent parents)	Refugee adolescent girls	10–16	Understand the SRH needs and risks of VYA in two humanitarian settings	Qualitative Cross-sectional FGDs (community mapping and photos)	There is a lack of SRH information. Schools, youth centers and religious institutions were identified as trustable places to obtain information There is a need for youth-directed programs and policies, involving peer-peer communication
16	Logie et al. Sexually transmitted infection testing awareness, uptake and diagnosis among urban refugee and displaced youth living in informal settlements in Kampala, Uganda: a cross-sectional study	2019	5 informal settlements in Kampala (Uganda)	445 (112 young men and 333 young women)	Youth self-identified as IDP or refugee	16–24	Explore factors associated with the STI services awareness, testing and diagnosis among urban refugees and displaced youth in Kampala, Uganda	Quantitative Cross-sectional Survey	The SRH stigma among urban refugee young women was associated with lower STI services awareness, testing uptake and diagnosis. Strategies tailored by gender and age can be promising for STI prevention
17	Logie et al. Social ecological factors associated with experiencing violence among urban refugee and displaced adolescent girls and young women in informal settlements in Kampala, Uganda: a cross-sectional study	2019	5 informal settlements in Kampala (Uganda)	333 (adolescent girls)	Adolescent girls and young women (AGYW) self-identified as IDP or refugee	16–24	Determine the prevalence of experience violence among urban refugees and displaced AGYW and the socio-demographic and social-ecological factors associated	Quantitative Cross-sectional Survey	The study provides information regarding the prevalence and correlates of young adulthood violence and recent intimate partner violence among urban refugee AGYW. There are a need for comprehensive interventions addressing economic and cultural gender-based inequities. Next studies should explore digital health technology use among urban refugee AGYW and its associations with risk for SGBV as well as its potential use in SGBV prevention
18	Logie et al. Exploring resource scarcity and contextual influences on wellbeing among young refugees in Bidi Bidi refugee settlement, Uganda: findings from a qualitative study	2021	Bidi Bidi refugee settlement (Uganda)	48 (24 men; 24 women)	Refugee or displaced adolescent and youth	16–24	Address knowledge gaps regarding Sexual and Gender-Based Violence (SGBV) risks among refugee adolescents and youth. The secondary aim (due to the emergence of COVID-19 during the study) was to explore experiences and perspectives toward COVID-19 among this population	Qualitative Cross-sectional FGDs and individual in-depth interviews (IDI)	Contextual factors affect refugee adolescents' and youth's well-being. The social-ecological model for SGBV among adolescent girls in humanitarian settings can be extended considering resource scarcity (water, food, firewood) and ecological contexts such as deforestation Strategies to address SGBV should be gender and age tailored
19	Logie et al. The role of context in shaping HIV testing and prevention engagement among urban refugee and displaced adolescents and youth in Kampala, Uganda: findings from a qualitative study	2021	Kampala’s informal settlements (Uganda)	44 (17 young men, 27 young women from Democratic Republic of Congo, Rwanda, Burundi and Sudan)	Refugee or displaced adolescent and youth	16–24	Understand experiences and access to HIV testing and prevention among urban refugee/displaced youth in Kampala	Qualitative Cross-sectional FGDs	The barriers to HIV testing and prevention were transportation costs to clinics, lack of private spaces due to overcrowded living conditions, low literacy, and language barriers. Symbolic contexts were medical mistrust and inequitable gender norms The interventions should include religious leaders to offer contextually relevant services and gender transformative approaches
20	Loutet et al. Sexual and reproductive health factors associated with child, early and forced marriage and partnerships among refugee youth in a humanitarian setting in Uganda: Mixed methods findings	2022	Bidi Bidi refugee settlement (Uganda)	In-depth interviews were conducted among 12 youth (boys and girls) and 8 elders aged 55 + years old who were sexual violence survivors, eight healthcare providers working in Bidi Bidi 48 youth participated in FGDs 120 youth answered the questionnaires	Refugee or displaced adolescent and youth	16–24	Address the gaps in the prevalence and health outcomes of the child, early and forced marriage (CEFMP) in humanitarian settings	Quantitative and Qualitative Cross-sectional Questionnaire FGDs and individual in-depth interviews (IDI)	CEFMP is common among youth in humanitarian settings and is influenced by poverty and education, impacting the reproductive outcomes among young refugee women. There is a need for context-specific interventions to address CEFMP
21	Malama et al. Factors associated with motherhood among urban refugee adolescent girls and young women in informal settlements in Kampala, Uganda	2022	5 informal settlements in Kampala (Uganda)	333 (adolescent girls)	Refugee or displaced adolescent girls and young women	16–24	Address the knowledge gap around the factors associated with motherhood among AGYW living in informal settlements	Quantitative Cross-sectional Survey	Motherhood among refugee AGYW was associated with food insecurity, depressive symptoms and recent contraceptive uptake. It is recommended an SRH and mental health integrated service as well as resource insecurity initiatives in the community
22	Marlow et al. The Sexual and Reproductive Health Context of an Internally Displaced Persons’ Camp in Northeastern Nigeria: Narratives of Girls and Young Women	2022	IDP camp in Northeastern Nigeria	25 (adolescent girls)	Single and married IDP adolescent girls and young women	15–24	Understand SRH AGYW's experiences as unwanted pregnancy, abortion, contraception, sexually transmitted infections (STIs), gender-based violence (GBV), and forced marriage	Qualitative Cross-sectional FGDs and individual in-depth interviews (IDI)	IDP adolescent girls and young women have poor SRH outcomes including unwanted pregnancies, STIs, GBV, and unsafe abortion. Due to the ongoing violence, food insecurity and lack of resources, AGYW are forced into sexual relationships and early marriages. Despite some SRH services available, AGYWs do not access them due to shame and stigma To improve poor SSR outcomes, it is necessary to provide integrated services that address the drivers of early sex and forced marriage
23	Marlow et al. Contraceptive use, menstrual resumption, and experience of pregnancy and birth among girls and young women in an internally displaced persons camp in Northeastern Nigeria	2022	IDP camp in Northeastern Nigeria	480 (adolescent girls)	Displaced adolescent girls and young women	15–24	Examine the relationships between contraceptive use, menstrual resumption, and pregnancy and birth experiences of AGYW in an IDP camp	Quantitative Cross-sectional Survey	Contraceptive services have yet to reach many AGYW in the IDP camps in Northeastern Nigeria
24	McMichael Unplanned but not unwanted? Teen pregnancy and parenthood among young people with refugee backgrounds	2013	Settlements in Victoria (Australia)	9 adolescent girls	Pregnant African-born young women with refugee backgrounds	11–19	Examines the ways adolescent girls with refugee backgrounds negotiate teen pregnancy and the challenges of early settlement	Qualitative Longitudinal (4 years) Field notes, open-ended survey questions and interviews	All adolescent reported an unplanned pregnancy, for someone the pregnancies it was unwanted. All of them quit the school, most of them reported they receive family support It is fundamental provide SRH education on the settlements, and for pregnant adolescent, programs should support them to return to school, work and ensure access to adequate housing
25	Meyer et al. Understanding the Sexual and Reproductive Health Experiences of Refugee and Host Community Adolescents and Youth in Rwanda During COVID-19: Needs, Barriers, and Opportunities	2022	Mahama Refugee Camp (Rwanda)	517 (adolescent girls and boys)	Refugee adolescent and youth	10–24	Understand the reasons for accessing SRH information and services by adolescents and youth refugees	Qualitative Cross-sectional FGDs and adolescents and youth refugees’ stories	Difficulties accessing SRH information and services (including stigmatization among service providers) were reported by adolescents and youth There is a need to increase specific SRH services prioritization for adolescents and youth in humanitarian settings
26	Odo et al. Sexual and Reproductive Health Needs and Problems of Internally Displaced Adolescents (IDAs) in Borno State, Nigeria: A Mixed Method Approach	2020	IDP camps in Borno State (Nigeria)	396 (220 adolescent boys and 176 adolescent women)	ID adolescent and youth	10–24	Identify the ID adolescents and youth SRH needs and problems and the strategies for improving their SRH	Quantitative and Qualitative Cross-sectional Questionnaire FGDs	SRH problems such as pregnancy complications, adolescent pregnancy, early sex experimentation, unsafe sex, maternal mortality, STIs, sexual harassment, genital fistulas, abortion and its complications were reported by internally displaced adolescents and youth Youth-friendly SRH services were reported as a possibility to address SSR needs and problems
27	Okanlawon et al. Contraceptive Use: Knowledge, Perceptions and Attitudes of Refugee Youths in Oru Refugee Camp, Nigeria	2010	Oru refugee camp (Nigeria)	280 youth (116 girls and boys)	Refugee AGYW	10–24	Examines the perceptions, beliefs, knowledge, and attitudes of refugee adolescents and youths toward contraceptive use and the access to them	Quantitative and Qualitative Cross-sectional Self-responded questionnaire FGDs and individual in-depth interviews (IDI)	There was a misinformation about contraceptives, perceived as a dangerous for women's health, and a difficult to accesses contraceptives The low contraception use resulted on a large number of unintended pregnancies and poor reproductive health outcomes The AGYW reported to engage in transactional sex and prostitution, highlighting their vulnerability and the need for specific policies for this population
28	Ortiz-Echevarria et al. Understanding the unique experiences, perspectives and sexual and reproductive health needs of very young adolescents: Somali refugees in Ethiopia	2017	Kobe refugee camp (Ethiopia)	126 (32 adults: adolescents' parents) and 94 adolescents (46 girls and 48 boys)	Somali refugee very young adolescents (VYA)	10–16	Understand the realities of very young adolescents (VYAs) in Kobe refugee camp, and their health needs, expectations, and goals	Qualitative Cross-sectional FGDs	VYA girls in Kobe refugee camp are at increased risk of poor SRH outcomes due to inequitable relations between boys and girls, risk of physical and sexual violence, early marriage and harmful traditional practices The next programs should reinforce positive behaviors for VYA improving SRH
29	Pandit et al. Constraints and current practices of menstrual hygiene among Rohingya adolescent girls	2022	Kutupalong refugee camp (Cox's Bazar, Bangladesh)	101 (adolescent girls)	Rohingya adolescent girls	13–18	Assess the MHM practices and constraints among adolescent girls in the Rohingya camps in Bangladesh	Quantitative and Qualitative Cross-sectional Semi-structured questionnaire FGDs	The Rohingya adolescent girls, have low premenstrual knowledge, face challenges regarding MHM as lack of disposable pads and inadequate toilets, exposing them to higher risk of sexual violence and live in limited supportive environments
30	Rakhshanda et al. Knowledge and practice regarding menstrual hygiene management among the Rohingya refugee adolescent girls in Cox’s Bazar, Bangladesh: a mixed method study	2021	Rohyngya refugee camp ( Cox's Bazar, Bangladesh)	340 adolescent girls (340 questionnaires and 7 IDI) 14 adolescents' mothers (2 FGD)	Rohingya adolescent girls	14–18	Understand the knowledge, practice and associated factors regarding MHM among adolescent girls in Rohingya refugee camps	Qualitative Cross-sectional Structured questionnaire Individual in-depth interviews (IDI)	The adolescents have not enough knowledge on menstruation and have not enough disposable pads. Distance to reach toilet, soap availability and sociocultural norms determines the cleanliness and frequency of change of pads There is a need for specific female’s toilets near to the homestead, with clean water and soap, available and affordable sanitary pads and MHM counselling
31	Soeiro et al. Period poverty: menstrual health hygiene issues among adolescent and young Venezuelan migrant women at the northwestern border of Brazil	2021	Boa Vista (Roraima State, Brasil)	153 (adolescent girls)	Venezuelan migrant AGYW	10–24	Provide an overview of the main MHM issues among Venezuelan migrant AGYW in Boa Vista, Roraima, Brazil	Quantitative Cross-sectional Self-responded questionnaire	The Venezuelan migrant AGYW have their MHM needs neglected and they were more affected due to the COVID-19 pandemic Efforts to address the MHM needs to be on collaboration NGO’s, UNHCR shelters and the Brazilian Government
32	Soeiro et al. A neglected population: Sexual and reproductive issues among adolescent and young Venezuelan migrant women at the northwestern border of Brazil	2021	Boa Vista (Roraima State, Brasil)	153 (adolescent girls)	Venezuelan migrant AGYW	10–24	Describe an overview of the main SRH issues affecting migrant Venezuelan AGYW in Boa Vista, Brazil	Quantitative Cross-sectional Self-responded questionnaire	The migrant Venezuelan AGYW in Boa Vista have their SRH needs neglected, and due to the COVID-19 pandemic they might be more affected. Efforts to address SRH for this population should be on a coordinate and comprehensive response among the Brazilian healthcare network and NGO's

**Table 2:** Evaluation of included studies

Qualitative studies	1.1	1.2	1.3	1.4	1.5
Asnong et al. 2018	Y	Y	N	Y	Y
Bol et al. 2021	Y	Y	Y	Y	Y
El Ayoubi et al. 2021	Y	Y	Y	Y	N
Elnakib et al. 2021	Y	Y	Y	N	N
Ghandour et al. 2021	Y	Y	Y	Y	Y
Kemigisha et al. 2020	Y	Y	Y	N	N
Knox 2017	Y	Y	N	Y	Y
Korri 2021	Y	Y	Y	Y	Y
Lee et al. 2017	Y	Y	Y	N	Y
Logie et al. 2020	Y	N	Y	Y	Y
Logie et al. 2021	Y	Y	Y	N	Y
Marlow et al. 2022	Y	Y	Y	Y	Y
McMichael 2013	Y	N	Y	Y	Y
Meyer et al. 2022	Y	Y	Y	Y	Y
Ortiz-Echevarria et al. 2017	N	Y	Y	Y	Y
Quantitative descriptive studies	4.1	4.2	4.3	4.4	4.5
Bakesiima et al. 2020	Y	Y	Y	Y	Y
Goers et al. 2022	Y	N	N	N	Y
Ganle et al. 2019	Y	Y	Y	Y	Y
Kågesten et al. 2017	Y	Y	Y	Y	Y
Logie et al. 2019	Y	Y	Y	N	Y
Logie et al. 2019	Y	Y	Y	N	Y
Malama et al. 2022	Y	Y	Y	N	Y
Marlow et al. 2022	Y	Y	N	Y	N
Soeiro et al. 2021	Y	Y	Y	N	Y
Soeiro et al. 2021	Y	Y	Y	N	Y
Mixed methods studies	5.1	5.2	5.3	5.4	5.5
Benner et al. 2010	N	N	Y	N	Y
Ivanova et al. 2019					
	Y	Y	Y	Y	Y
Loutet. et al. 2022	N	Y	Y	Y	Y
Odo et al. 2020	Y	Y	Y	Y	Y
Okanlawon et al. 2010	N	N	Y	Y	Y
Pandit et al. 2022	N	Y	N	Y	Y
Rakhshanda et al	Y	Y	Y	Y	Y

## Results

The results were analyzed using a narrative approach and thematic analysis of the original quotations [20, 21]. Disagreements were resolved through discussion until a consensus was reached. After reviewing all of the literature, seven categories emerged [52]: adolescent pregnancy, contraceptive use and access, menstrual hygiene management (MHM), sexually transmitted infections (STI) and HIV, SRH information/knowledge/access, overview of the main SRH needs, other issues related to SRH (sexual and gender-based violence and child, early and forced marriage or partnership).

### Overview of the studies

Among 1290 studies screened by abstracts, 32 met the eligibility criteria; 15 were qualitative studies [22–36], 10 were quantitative [6, 37–45] and seven were mixed-methods studies [46–52]. The PRISMA flowchart of included studies is shown in Fig. 1.

More than 50% [6, 9, 22–24, 26, 27, 29, 31–39, 41–44, 46, 49–52], 12 studies were published between 2017 and 2020 [21, 25, 26, 28, 32, 35, 36, 38, 39, 38–47], one was published in 2013 [30] and two in 2010 [44, 48].

There were 20 studies with female participants only [9, 22–26, 29, 30, 33–36, 38, 40–43, 49, 50], and 12 studies with female and male participants [22, 25, 28–30, 32, 40, 46, 48, 50]. Parents, non-governmental organizations workers, and health workers were interviewed regarding their opinion of the AGYW's SRH issues. [22, 30, 33, 36, 48, 52].

Most of the studies were performed in African countries: nine in Uganda [32, 35, 37, 40–42, 47, 48, 52], four in Nigeria [26, 43, 49, 50], two in Ethiopia [30, 31], one in Egypt [33], one in Ghana [38], and one in Rwanda [29]. The remainder were: three studies were from Thailand [22, 25, 46], three from Lebanon [23, 24, 36], one from Iraq [45], two from Bangladesh [51, 52], one from Jordan [34], two from Brazil [6, 44], one from Australia [27] and one study was performed in two different countries (Thailand and Ethiopia) [39].

### Adolescent pregnancy

Four studies reported specifically on adolescent pregnancy: two of them were quantitative [31, 42], and the other two were qualitative [22, 27].

The studies were conducted with refugee AGYW in refugee camps or informal settlements on the Thailand-Myanmar border [22], Uganda [42], Southwest Ethiopia [31], and Australia [27]. The overall prevalence of pregnancy among AGYW was 16% for the study conducted in Australia [27], 21.7% in Uganda [42] and 23% in Ethiopia [31], and more than 50% had no formal or primary education [22, 31, 42]. In the qualitative studies [22, 27], all AGYW students dropped out of school after becoming pregnant. Access to and utilization of maternal health services by pregnant AGYW were not investigated in any of these studies.

The two qualitative studies [21, 30] interviewed specific pregnant women. Asnong et al. (two refugee camps in Thailand-Myanmar border) [21] reported three adolescents and young women were forced to marry after getting pregnant, and only two pregnant young women over 20 said they stopped contraception intending to get pregnant. In both qualitative studies, all the adolescents quit school: some were too ashamed to continue studying, others were expelled because of pregnancy, and others due to motherhood responsibilities.

The adolescents also complained about social isolation due to the burden of taking care of a child and doing all of the housework.

The two quantitative studies reported a pregnancy prevalence of 21.7 (five informal settlements in Kampala,Uganda) [41] and 23% (refugee camp in Ethiopia) [30]. Bol et al. [31] described poor SRH knowledge (40%), low prevalence of contraceptive use (6.5%), and low educational status (42% with no formal education). The pregnancy rates among adolescent girls and young women with no formal education were 3.4 times higher than girls who attended secondary school and above.

Regarding access to and utilization of maternal health services, all pregnant AGYW in the refugee communities on the Thailand-Myanmar border were attending ANC as the study was conducted in the ANC clinics [22]. The other three studies did not report on that.

Malama et al. (Uganda) [41] reported AGYW who are mothers were twice as likely to experience food insecurity and depressive symptoms.

All studies reported financial difficulties, including among the married subjects, and few of the pregnant, unmarried AGYW had received family support.

### Contraceptive use and access

Four studies reported contraceptive use and access, three quantitative [37, 38, 43] and one mixed method [50].

The studies were performed in refugee camps in Northern Uganda [37], Ghana [38], Northeastern [43] and Southwest Nigeria [50]. They addressed the use and access to modern contraceptives and the cultural and religious beliefs for not using them.

All the studies reported very low use of modern contraceptives: 8% (Nigeria) [43], 8.7% (Uganda) [37], and 11,7% [38] for the quantitative studies [37, 38, 43] and 32% for the mixed-method study [50]). The method used by the AGYW varied among the studies: injectable contraceptive (42%), oral contraceptive (5.5–29%), male condom (16–55%), implant (36%), emergency contraception (18%).

The reasons reported for not using contraceptives were difficulties accessing health services or lack of information about health services: Ganle et al. [38] reported 39% of AGYW (refugee camp, Ghana) who heard about contraception did not know where they could get it, and Okanlawon et al. (refugee camp, Nigeria) [50] reported 60% did not use contraception due fear of side effects (39–80%); cultural beliefs (11–17%); and partner prohibition (16–40%).

The studies also reported misinformation regarding contraception: Okanlawon et al. [50] described a belief that AGYW would become infertile after contraception use, and 84% of the AGYW in the Ganle et al. [38] study believed that the women who use contraceptives become promiscuous.

### Menstrual hygiene management (MHM)

Five studies reported on MHM, two quantitative [39, 44], one qualitative [35] and two mixed methods [51, 52]. They were performed in refugee camps or migrant settlements: Venezuelan migrant settlements in the North-western border of Venezuela-Brazil [44], refugee settlements in Cox's Bazar Bangladesh [51, 52], refugee settlement in Uganda [35], Kobe refugee camp in Ethiopia, and Myanmar migrant communities in Thailand [39], and all described a poor MHM: distance to reach toilets, lack of water, shortage of menstrual hygiene supplies and lack of knowledge about menstruation.

The studies reported there was no private toilet for the majority of the AGYW to use (85–93%) [39, 51], and a lack of MHM products such as hygienic pads (disposable or reusable), soap and clean water (55–83%) [39, 44, 51]. In the Venezuelan migrant settlements in the North-western border of Venezuela-Brazil and in the refugee settlements in Cox's Bazar Bangladesh there was no proper place to wash or dry the disposable pads (65%) [44, 51].

The AGYW reported feeling embarrassed during menstruation (50–60%) [35, 44, 51] and missing school because of MHM issues (refugee settlement in Uganda) [35].

The study performed in the Rohingya camp reported that 72% of AGYW were not allowed to go out during the menstruation period, and 88% were not allowed to cook [51].

The studies also reported that the toilets in the internally displaced people (IDP) camps were distant, and the AGYW (55–65%) feared going to the toilet because of the risk of sexual violence [35, 44, 51].

### Sexually transmitted infections (STI) and HIV

Two studies reported on STI [40] and HIV [32]. Both were conducted with urban refugees and displaced youth living in informal settlements in Kampala, Uganda. They described there is a stigma associated with STI and HIV testing.

The study on STIs [39] described 74% had never been tested for STIs and more than half of the adolescents (56%) were unaware of the STI testing services. Among those tested, 16% did not know to inform the test results received. The STIs reported by the adolescents were: 10% more than two STIs, 15% herpes, 9% gonorrhea, and 6% syphilis. There was no information regarding HIV.

Among AGYW, stigma on STI testing was associated with lower contraceptive use and food insecurity [39].

The HIV study evidenced the barriers to HIV testing and prevention are the cost of transportation to HIV testing services, language barriers, lack of private spaces to do the self-test, medical mistrust, and inequitable gender norms [34].

### SRH information/knowledge/access

Seven studies reported on SRH information, knowledge and access: five qualitative [23–25, 29, 34] and two mixed methods [46, 47]. The qualitative studies were conducted in Rwanda (Mahama camp and in the surrounding host community) [29], in a town in Lebanon’s Bekaa governorate (Syrian Refugee Adolescent Girls) [23], in the West Bank and Jordan (Palestinian refugee camps) [34], in an industrial area in North-East Beirut in Lebanon (Syrian refugee adolescent girls) [24], in Thailand border with Myanmar (one migrant community and one Refugee camp) [25]; the mixed-methods study were conducted in villages along the northwestern border inside Thailand (two refugee camps with young Burmese refugees) [46] and in Nakivale refugee settlement, Uganda (migrants from DR Congo and Burundi) [47]. Overall, there is a lack of SRH information and misconceptions, and an unsatisfactory number of SRH services.

Three studies identified family members, mostly mothers, as trusted sources of SRH information. [23, 24, 47].

Schools were also described as a place to obtain SRH information, although most AGYW did not attend school. [23, 25, 29, 47].

Regarding SRH knowledge, in the Nakivale refugee settlement, Uganda, Ivavona et al. [47] reported that 16% of the AGYW did not know about STIs and 52% were able to mention only one STI; 14% did not know about contraception, 44% knew one method and 15% knew three or more methods.

Benner et al. [46] identified that 66% of Burmese refugees AGYW in Thailand refugee camps did not know if it was possible to get pregnant after the first sexual intercourse, 68% did not know if women can take contraceptive pills daily, 59% did not know if condoms can be used during sex, and 45% did not know if it is accceptable for a boy to sometimes force a girl to have sex if he loves her.

Meyer et al. [29] described the decrease in SRH information sessions and SRH services after the onset of the COVID-19 pandemic in Rwanda refugee community; the AGYW complained of unplanned pregnancies due to the contraception disruptions and about the suspension of the SRH activities from local Non-Governmental Organizations (NGOs).

All studies highlighted the need for shaping programs by sex and age to address SRH information and recommended empowering adolescent mothers as agents of SRH evidence-based information.

### Overview of the main SRH needs

Four studies, two qualitative [26, 30], one quantitative [6] and one mixed methods [49] reported an overview of SRH information, needs, access to it, and outcomes. The qualitative studies were conducted in IDP camps in Nigeria [26] and a refugee camp in Ethiopia (Somali adolescent girls) [30]; the quantitative study was conducted in Venezuelan migrant settlements on the Northwestern border of Venezuela and Brazil [6]; and the mixed-methods studies were conducted in IDP camps in Borno State (Nigeria). [49].

All of them evidence poor SRH outcomes as limited access to family planning, unsafe sex, early marriages, pregnancy complications and low knowledge and access to SRH services.

Soeiro et al. [6] reported that, among Venezuelan pregnant AGYW (in migrant settlements on the Northwestern border of Venezuela and Brazil), 33% were not attending ANC, and the reasons were not knowing where to go (40%), difficulty reaching the health center (20%) and not having personal documents (20%). The main self-reported SRH concerns were contraception (35%), and 75% of the adolescents who went to a health center did not get it.

In the Odo et al. [49] study, 98% of adolescent girls (IDP camps, Nigeria) described sex education as important, and they agreed that the main SRH problems were teenage pregnancy (72%), early marriage (76%), menstrual problems (70%), and maternal mortality (80%).

Marlow et al. [26] also demonstrate that food insecurity in the IDP camp (Nigeria) has driven adolescent girls to sex in exchange for goods or into forced marriages.

All articles highlighted the need for specific SRH services for IDP, refugees or migrant AGYW.

### Other issues related to SRH

#### Child early and forced marriage or partnership (CEFMP)

Four studies one quantitative [45], two qualitative [33, 36] and one mixed methods [48] reported on CEFMP (formal marriage or an informal union, before reaching the age of 18) [53]. The quantitative study was conducted in the Kurdistan Region of Iraq (IDPs AGYW) [45]. The qualitative study was conducted in a Palestinian refugee camp [36] and in three governorates in Egypt (Syrian refugees AGYW) [33], and the mixed-methods study was conducted in a refugee settlement in Uganda (South Sudanese AGYW) [48].

In the countries where the studies were conducted, the minimum age of marriage for girls is 18 in Egypt, Iraq, and Uganda, and 17 in Palestine. In Syria and South Sudan (AGYW origin countries), the minimum age of marriage is 18 for Syrian girls, and there is no minimum age of marriage for South Sudanese girls [54]. The four studies had an association between social and financial insecurity and early marriage.

Adolescent marriage was associated with negative SRH outcomes such as lack of family planning, unplanned pregnancies, and disruption of schooling [33, 36, 45, 48]. Elnakib et al. (Syrian refugees AGYW, in Egypt) [33] described adolescent girls' isolation after marriage and difficulties with their baby birth registration.

Goers et al. (IDP AGYW in the Kurdistan Region of Iraq) [45] described that 38% of the AGYW [10–19] were married. The risk of marriage before 18 years was 6.2 times higher for girls than boys. Only 6% of the married or engaged refugee adolescent girls were in school. The influencing factors in marriage decisions were displacement (12%), money/resources (21%) and war/conflict (29%).

In the Loutet et al. (South-Sudanese AGYW in Uganda) [48] study, 75% of AGYW were married, and 57% had primary-level education or lower. CEFMP was associated with forced pregnancy (50%), forced abortion (45%), missed school due to sexual violence (95%) and survival sex work (64%).

In contrast to the other three studies, the findings of Knox et al. (Palestine refugee camp) [36] demonstrated that not all child marriages were forced. Some AGYW reported that they felt they were a burden to their families due to post-conflict economic crises and chose to marry.

#### Sexual and gender-based violence (SGBV)

Two studies reported SGBV as the main outcome of their research. The quantitative study was conducted in informal settlements in Kampala, Uganda (refugee AGYW were from South Sudan, DR Congo, Burundi and Rwanda); the qualitative study was conducted in a refugee camp in the Yumbe District, Uganda (refugee AGYW were from South Sudan and DR Congo).

In a quantitative study, Logie et al. [41] evidenced that over half of the participants (54%) reported intimate partner violence (IPV) in the last 12 months (55% reported polyvictimization: physical, sexual, and control violence). IPV polyvictimization was associated with depressive symptoms (90%), and food insecurity (94%).

In a qualitative study, Logie et al. [28] explored the refugee adolescent well-being factors and SGBV was associated with poverty, food insecurity, and unemployment, leading to CEFMP and transactional sex. The study also described how deforestation exacerbated sexual violence, as the AGYW must go further to collect water and firewood.

The studies did not report which resources were available to address SGBV in the research settings.

## Discussion

This review aimed to explore the current qualitative and quantitative research landscape on the SRH needs of adolescent girls and young women displaced by humanitarian crises.

Specific studies targeting SRH of AGYW migrants (10–24 years old) are recent, with a significant increase since 2021. Possible explanations for this increase could be that the forcibly displaced population has quintupled in the last two decades, from 20.7 million in 2000 to 100 million in 2022 [55], and that adolescent health, including SRH, has been included in the 2030 Sustainable Development Goals since 2015 [3].

The reviewed studies reported greater pregnancy rates before age 18, ranging from 16% (migrant settlements in Australia) [27] to 23% (refugee camps in Ethiopia) [31], compared with the global pregnancy rate before age 18 (14%) [56]. They also reported that 100% of the adolescents had quit school, more than twice reported in a study conducted with pregnant AGYW in Cameroon (41.6% drop-off rate). [57].

Regarding modern contraceptive use, 43% of AGYW in low- and middle-income countries use male condoms, oral pills or injectables [58], compared with 8–32% [50] of contraceptive use in the presented studies.

The CEFMP has been described as a cultural and societal pattern in some countries [59]. However, in the countries where the studies were conducted, the minimum age of marriage for girls was 17 years. The analyzed studies corroborate that in humanitarian settings, the situation of insecurity, increased poverty and often the loss of family members lead to girls being more likely to engage in CEFMP. [33, 45, 48]. Recommendations on how to address it do not include humanitarian settings [55, 56].

Furthermore, CEFMP is also linked to lack of contraception [60] and adolescent pregnancies [9, 59], as well as. Pregnancy and childbirth complications are one of the leading causes of death among 15- to 19-year-old girls worldwide [9, 60]. Studies in this review showed a high prevalence of adolescent pregnancies, frequently unplanned [22, 27, 31, 42], but most of them did not discuss those pregnant adolescent's morbidity and mortality rate in the study settings. As there is a lack of health services and proper data systems records in humanitarian settings, the impact of those pregnancies probably remains underestimated. [10].

The global increase of STIs among adolescents has been described for boys and girls. However, the prevalence is higher among adolescent girls [61]. New HIV infections are also higher among adolescent girls [62]. Logie et al. [32, 40] identified barriers to HIV and STI testing and stigma among adolescent refugee girls living in Kampala, Uganda, hindering these diagnoses among AGYW. There are few studies on STIs and HIV among migrant AGYW; a study conducted in South Africa also reported difficulty accessing health services as a risk factor for HIV [63].

Concerning access to SRH services, the findings evidence a lack of these services in humanitarian contexts [46, 47], which have been further affected by the COVID-19 pandemic. There are scarce studies on this topic and even fewer for migrant AGYW. A systematic review conducted in low- and middle-income countries also reported the impact of the COVID-19 pandemic on the AGYW's SRH due to the interruption of SRH services, resulting in increased rates of early marriage, sexual or intimate partner violence, and disruption of maternal care [64]. Meyer et al. [29] described unplanned pregnancies among refugee AGYW in Rwanda due to contraception disruptions during this period.

Most studies also recommended specific SRH programs and health services for adolescents in humanitarian settings. However, they did not report which interventions were available in the studies' countries. Notwithstanding the increasing number of studies on this subject, specific interventions targeting SRH for adolescent girls are still uncommon. A systematic review [65] published in 2019 identified only nine SRH interventions for adolescents and youth in armed conflict settings. Only one study was published before 2012 (in 2006), and the majority were implemented in African countries, one in Colombia and one in Pakistan, evidencing the need for global SRH interventions targeting AGYW.

This systematic review evaluated qualitative, quantitative and mixed-methods studies on SRH issues of AGYW displaced by humanitarian crises in African, Asia and South American countries. All issues reported in the studies are included in the SDG targets. There are some strategies and tools described to address the SRH of AGYW in humanitarian contexts [7, 66]. However, the studies did not mention these or any other strategies to improve SRH in the study settings. In addition, data collection, monitoring and evaluation in these contexts still need to be standardized to understand the gaps better and adapt specific interventions in to reach the SDGs 2023.

## Limitations

We included studies with girls and young women from 10 to 24 years old, and we may have missed data from studies which included all women of reproductive age. Moreover, as we considered studies in English, Spanish and French, we may have missed some studies in other languages and some reports from grey literature.

## Conclusions

The SRH of adolescent girls and young women in humanitarian crisis contexts has been neglected. Despite the growing number of studies on this population (32 studies were analyzed, and most of them were published between 2020 and 2022), there was no description of local interventions to address the reported issues. The migrant AGYW have difficulty accessing contraceptives, a high prevalence of unplanned pregnancies, child marriage, and sexual and gender-based violence. Improving data collection, monitoring, and evaluation may help humanitarian support and researchers to establish specific interventions for this population.

### Supplementary Information

**Additional file 1. **Search strategy.

**Additional file 2.** Mixed methods appraisal tool (MMAT) version 2018.

## Data Availability

The datasets used and/or analyzed during the current study are available from the corresponding author upon reasonable request.

## Abbreviations

AGYW: Adolescent girls and young women
ANC: Antenatal care
CEFMP: Child, early and forced marriage or partnership
COVID-19: Coronavirus Disease 2019
FGD: Focal Groups Discussion
HIV: Human Immunodeficiency Virus
IDI: In-depth interviews
IDP: Internally displaced population
IPV: Intimate partner violence
KRI: Kurdistan Region of Iraq
MeSH: Medical Subject Headings
MHM: Menstrual hygiene management
MMAT: Mixed Methods Appraisal Tool
NGO: Non-Governmental Organization
PRISMA: Preferred Reporting Items for Systematic Reviews and Meta-Analyses
SDG: Sustainable Development Goals
SGBV: Sexual and Gender Based Violence
SRH: Sexual and reproductive health
STI: Sexually transmitted infections
UNFPA: United Nations Funds for Population
UNHCR: United Nations High Commissioner for Refugees
VYA: Very young adolescents
WHO: World Health Organization
