# Supplementary material for: Sexual and reproductive health (SRH) needs for forcibly displaced adolescent girls and young women (10–24 years old) in humanitarian settings: a mixed-methods systematic review
Source: Reprod Health. 2023 Nov 24;20:174. doi: 10.1186/s12978-023-01715-8 (PMC10668438; doi:10.1186/s12978-023-01715-8)
Supplement: Supplementary file 1 — Additional file 1. Search strategy. [file 12978_2023_1715_MOESM1_ESM.docx]

| Descritor em português: | **Adolescente** |
| --- | --- |
| Descritor em inglês: | **Adolescent** |
| Descritor em espanhol: | **Adolescente**  [Espanhol da Espanha](https://decs.bvsalud.org/ths/resource/?id=29315&filter=ths_termall&q=adolescente#descriptorSpanish) |
| Descritor em francês: | **Adolescent** |
| Termo(s) alternativo(s): | Adolescentes Adolescência Jovem Jovens Juventude |
| Código(s) hierárquico(s): | M01.060.057 |
| Identificador Único RDF: | [https://id.nlm.nih.gov/mesh/D000293](https://id.nlm.nih.gov/mesh/D000293.html) |
| Nota de escopo: | Pessoa com 13 a 18 anos de idade. |

| MESH | Adolescent OR Adolescents OR Adolescence OR Teens OR Teen OR Teenagers OR Teenager OR Youth OR Youths OR "Adolescents, Female" OR "Adolescent, Female" OR "Female Adolescent" OR "Female Adolescents" |
| --- | --- |

DESCRITORES:

| Descritor em português: | **Vulnerabilidade em Saúde** |
| --- | --- |
| Descritor em inglês: | **Health Vulnerability** |
| Descritor em espanhol: | **Vulnerabilidad en Salud**  [Espanhol da Espanha](https://decs.bvsalud.org/ths/resource/?id=50263&filter=ths_termall&q=vulnerabilidade#descriptorSpanish) |
| Descritor em francês: | **Vulnérabilité en Santé** |
| Termo(s) alternativo(s): | Vulnerabilidade e Saúde |
| Código(s) hierárquico(s): | SP2.070.315.850 |
| Identificador DeCS: | 50263 |
| ID do descritor: | DDCS050263 |
| Documentos indexados na Biblioteca Virtual em Saúde (BVS): | [Clique aqui para acessar os documentos da BVS](https://pesquisa.bvsalud.org/portal/?q=mh:(%22Vulnerabilidade%20em%20Sa%C3%BAde%22)) |

| NÃO É MESH | "Health Vulnerability" |
| --- | --- |

OR

| Descritor em português: | **Vulnerabilidade Sexual** |
| --- | --- |
| Descritor em inglês: | **Sexual Vulnerability** |
| Descritor em espanhol: | **Vulnerabilidad Sexual**  [Espanhol da Espanha](https://decs.bvsalud.org/ths/resource/?id=57434&filter=ths_termall&q=vulnerabilidade#descriptorSpanish) |
| Descritor em francês: | **Vulnérabilité Sexuelle** |
| Código(s) hierárquico(s): | SP2.070.315.850.698 SP2.070.578.468.655.822 SP3.311.900.686.789 |
| Nota de escopo: | Estado em que os aspectos individuais e sociais relacionados às desigualdades de gênero tornam indivíduos mais sujeitos à vitimização por violência ou pela falta de acesso aos direitos humanos. (Adaptado de: https://www.arca.fiocruz.br/handle/icict/33545) |

| NÃO É MESH | "Sexual Vulnerability" |
| --- | --- |

OR

| Descritor em português: | **Saúde Sexual** |
| --- | --- |
| Descritor em inglês: | **Sexual Health** |
| Descritor em espanhol: | **Salud Sexual**  [Espanhol da Espanha](https://decs.bvsalud.org/ths/resource/?id=54864&filter=ths_termall&q=SAUDE%20SEXUAL#descriptorSpanish) |
| Descritor em francês: | **Santé sexuelle** |
| Código(s) hierárquico(s): | N01.400.663 SP2.770.913 |
| Identificador Único RDF: | [https://id.nlm.nih.gov/mesh/D000074384](https://id.nlm.nih.gov/mesh/D000074384.html) |
| Nota de escopo: | Estado de bem-estar físico, emocional, mental e social em relação à SEXUALIDADE, de acordo com a Organização Mundial da Saúde. |

| MESH | "Sexual Health" OR "Health, Sexual" |
| --- | --- |

OR

| Descritor em português: | **Saúde Reprodutiva** |
| --- | --- |
| Descritor em inglês: | **Reproductive Health** |
| Descritor em espanhol: | **Salud Reproductiva**  [Espanhol da Espanha](https://decs.bvsalud.org/ths/resource/?id=54845&filter=ths_termall&q=SAUDE%20SEXUAL#descriptorSpanish) |
| Descritor em francês: | **Santé reproductive** |
| Termo(s) alternativo(s): | Saúde Sexual e Reprodutiva |
| Código(s) hierárquico(s): | N01.400.625 SP2.840.318 |
| Identificador Único RDF: | [https://id.nlm.nih.gov/mesh/D060728](https://id.nlm.nih.gov/mesh/D060728.html) |
| Nota de escopo: | 1) O estado do SISTEMA REPRODUTOR ao funcionar de forma ótima sem evidência de doença, distúrbios ou deficiências. 2) Estado de completo bem-estar físico, mental e social, e não de mera ausência de doença, em todos os aspectos relativos ao sistema reprodutivo, suas funções e processos, em todas as fases da vida. Por esse motivo, a saúde reprodutiva implica que as pessoas sejam capazes de ter um a vida sexual responsável, satisfatória e segura e que elas tenham a capacidade de se reproduzir e a liberdade de decidir se, quando e com que frequência fazê-lo. Implícitos estão o direito de homens e mulheres serem informados e de terem acesso a método de regulação da fertilidade de sua escolha, que sejam seguros, eficazes, acessíveis e aceitáveis, e o direito ao acesso a serviços de saúde adequados que permitam que as mulheres sigam com segurança durante a gravidez e o parto e que ofereçam aos casais todo o necessário para terem um bebê saudável. (Tradução livre do original: WHO. Health topics - Reproductive Health. Disponível em http://www.who.int/topics/reproductive_health/en/) |

| MESH | "Reproductive Health" OR "Health, Reproductive" |
| --- | --- |

AND

| Descritor em português: | **Refugiados** |
| --- | --- |
| Descritor em inglês: | **Refugees** |
| Descritor em espanhol: | **Refugiados**  [Espanhol da Espanha](https://decs.bvsalud.org/ths/resource/?id=12458&filter=ths_termall&q=REFUGIADO#descriptorSpanish) |
| Descritor em francês: | **Réfugiés** |
| Termo(s) alternativo(s): | Desalojados Deslocados Pessoas Desalojadas Pessoas Deslocadas Pessoas Deslocadas Internamente População Desalojada População Deslocada População Evacuada Refugiado Acolhido pela Convenção Refugiado Acolhido pelo Protocolo Refugiado Acolhido por Convenção Refugiado Acolhido por Mandato Refugiado Reconhecido Refugiado Retornado Refugiado a Posteriori Refugiado da Convenção Refugiado de Direito Refugiado de Fato Refugiados Políticos Requerentes de Asilo Requerentes de Asilo Político |
| Código(s) hierárquico(s): | M01.755 SP3.522.561.200.735 SP8.665.912.703 |
| Identificador Único RDF: | [https://id.nlm.nih.gov/mesh/D012036](https://id.nlm.nih.gov/mesh/D012036.html) |
| Nota de escopo: | 1. Pessoas que fogem para um lugar seguro, especialmente aquelas que fogem para um outro país ou poder para escapar de perigo, perseguição ou dificuldade econômica em seu próprio país ou residência habitual. 2. Migrantes involuntários, vítimas de condições políticas, guerras ou catástrofes naturais. (Tradução livre do original: Popline, 2002) 3. Requerente de asilo: Pessoa que pretende ser admitida num país como refugiado e que aguarda uma decisão relativamente ao seu requerimento para obter o estatuto de refugiado segundo os instrumentos, internacionais e nacionais, competentes. Em caso de indeferimento, tem que abandonar o país e poderá ser expulsa, tal como qualquer estrangeiro em situação irregular, excepto se for autorizado a permanecer por razões humanitárias ou outros fundamentos relacionados. (Fonte: Glossário sobre Migração. Organização Internacional para as Migrações. Genebra. 2009. Disponível em: http://publications.iom.int/system/files/pdf/iml22.pdf) |

| MESH | Refugees OR Refugee OR "Political Asylum Seekers" OR "Asylum Seeker, Political" OR "Asylum Seekers, Political" OR "Political Asylum Seeker" OR "Seekers, Political Asylum" OR "Political Refugees" OR "Political Refugee" OR "Refugee, Political" OR "Refugees, Political" OR "Asylum Seekers" OR "Asylum Seeker" OR "Seeker, Asylum" OR "Seekers, Asylum" OR "Displaced Persons" OR "Displaced Person" OR "Person, Displaced" OR "Persons, Displaced" OR "Internally Displaced Persons" OR "Displaced Person, Internally" OR "Displaced Persons, Internally" OR "Internally Displaced Person" |
| --- | --- |

OR

| Descritor em português: | **Migrantes** |
| --- | --- |
| Descritor em inglês: | **Transients and Migrants** |
| Descritor em espanhol: | **Migrantes**  [Espanhol da Espanha](https://decs.bvsalud.org/ths/resource/?id=14563&filter=ths_termall&q=Transients%20and%20Migrants#descriptorSpanish) |
| Descritor em francês: | **Population de passage et migrants** |
| Termo(s) alternativo(s): | Migrante Nomadismo Não-Migrante Não-Migrantes Nômade Nômades Posseiro Posseiros Trabalhador Migrante Trabalhadores Migrantes Transeunte Transeuntes Transeuntes e Migrantes |
| Código(s) hierárquico(s): | M01.920 SP3.522.561.200.504 SP8.665.912.601 |
| Identificador Único RDF: | [https://id.nlm.nih.gov/mesh/D014171](https://id.nlm.nih.gov/mesh/D014171.html) |
| Nota de escopo: | 1. Pessoas que frequentemente mudam seu lugar de residência. 2. Nômade: Modo de vida tradicional de certos povos rurais que não vivem continuadamente na mesma área, mas se mudam de maneira cíclica ou periódica, geralmente em busca de terras para plantar ou caçar e lugares que tenham água, estando bem adaptados à mudança de ambiente. (Tradução livre do original: Material V - Gunn, S.W.A. Multilingual Dictionary of Disaster Medicine and International Relief, 1990) |

| MESH | "Transients and Migrants" OR "Migrants and Transients" OR Migrants OR Migrant |
| --- | --- |

**DATABASE**

**https://www.fcm.unicamp.br/fcm/biblioteca-fcm/bases-em-saude-sbe**

| **Fonte** | **Vocabulário de Assuntos** | DESCRITORES E TERMOS LIVRES UTILIZADOS NA ESTRATÉGIA DE BUSCA | | | | |
| --- | --- | --- | --- | --- | --- | --- |
|  |  | 1 | 2 | 3 | 4 (TERMO LIVRE) | 5 |
| **PUBMED** | MeSH - Medical Subject Headings | **Adolescent** | "Health Vulnerability" | "Sexual Vulnerability" | "Sexual Health" | "Reproductive Health" |
| **PUBMED**  **PMC** | MeSH - Medical Subject Headings | **Adolescent** | "Health Vulnerability" | "Sexual Vulnerability" | "Sexual Health" | "Reproductive Health" |
| **Scopus** |  | **Adolescent** | "Health Vulnerability" | "Sexual Vulnerability" | "Sexual Health" | "Reproductive Health" |
| **WEB OF SCIENCE** |  | **Adolescent** | "Health Vulnerability" | "Sexual Vulnerability" | "Sexual Health" | "Reproductive Health" |
| **EMBASE** | Emtree | **Adolescent** | "Health Vulnerability" | "Sexual Vulnerability" | "Sexual Health" | "Reproductive Health" |

**DATABASE**

**https://www.fcm.unicamp.br/fcm/biblioteca-fcm/bases-em-saude-sbe**

| **Fonte** | **Vocabulário de Assuntos** | DESCRITORES E TERMOS LIVRES UTILIZADOS NA ESTRATÉGIA DE BUSCA | | |
| --- | --- | --- | --- | --- |
|  |  |  |  |  |
| **PUBMED** | MeSH - Medical Subject Headings | Refugees | "Transients and Migrants" |  |
| **PUBMED**  **PMC** | MeSH - Medical Subject Headings | Refugees | "Transients and Migrants" |  |
| **Scopus** |  | Refugees | "Transients and Migrants" |  |
| **WEB OF SCIENCE** |  | Refugees | "Transients and Migrants" |  |
| **EMBASE** | Emtree | Refugees | **transients and migrants**  **use:**  **migration** |  |

**Search Strategy**

**Adolescent AND ("Health Vulnerability" OR "Sexual Vulnerability" OR "Sexual Health" OR "Reproductive Health") AND (Refugees OR "Transients and Migrants")**

**Search Strategy**

| **Fonte** | **Estratégia** | **Nº de Artigos** | **Data** |
| --- | --- | --- | --- |
| **PUBMED** | ((Adolescent OR Adolescents OR Adolescence OR Teens OR Teen OR Teenagers OR Teenager OR Youth OR Youths OR "Adolescents, Female" OR "Adolescent, Female" OR "Female Adolescent" OR "Female Adolescents") AND (((("Health Vulnerability") OR ("Sexual Vulnerability")) OR ((Sexual Health[MeSH Terms]) OR ("Sexual Health"[Title/Abstract] OR "Health, Sexual"[Title/Abstract]))) OR ((Reproductive Health[MeSH Terms]) OR ("Reproductive Health"[Title/Abstract] OR "Health, Reproductive"[Title/Abstract])))) AND (((Refugees[MeSH Terms]) OR (Refugees[Title/Abstract] OR Refugee[Title/Abstract] OR "Political Asylum Seekers"[Title/Abstract] OR "Asylum Seeker, Political"[Title/Abstract] OR "Asylum Seekers, Political"[Title/Abstract] OR "Political Asylum Seeker"[Title/Abstract] OR "Seekers, Political Asylum"[Title/Abstract] OR "Political Refugees"[Title/Abstract] OR "Political Refugee"[Title/Abstract] OR "Refugee, Political"[Title/Abstract] OR "Refugees, Political"[Title/Abstract] OR "Asylum Seekers"[Title/Abstract] OR "Asylum Seeker"[Title/Abstract] OR "Seeker, Asylum"[Title/Abstract] OR "Seekers, Asylum"[Title/Abstract] OR "Displaced Persons"[Title/Abstract] OR "Displaced Person"[Title/Abstract] OR "Person, Displaced"[Title/Abstract] OR "Persons, Displaced"[Title/Abstract] OR "Internally Displaced Persons"[Title/Abstract] OR "Displaced Person, Internally"[Title/Abstract] OR "Displaced Persons, Internally"[Title/Abstract] OR "Internally Displaced Person"[Title/Abstract])) OR ((Transients and Migrants[MeSH Terms]) OR ("Transients and Migrants"[Title/Abstract] OR "Migrants and Transients"[Title/Abstract] OR Migrants[Title/Abstract] OR Migrant[Title/Abstract]))) | 331 | 05/01/2023 |
| **PUBMED**  **PMC** | ((Adolescent OR Adolescents OR Adolescence OR Teens OR Teen OR Teenagers OR Teenager OR Youth OR Youths OR "Adolescents, Female" OR "Adolescent, Female" OR "Female Adolescent" OR "Female Adolescents") AND (((("Health Vulnerability") OR ("Sexual Vulnerability")) OR ((Sexual Health[MeSH Terms]) OR ("Sexual Health"[Title/Abstract] OR "Health, Sexual"[Title/Abstract]))) OR ((Reproductive Health[MeSH Terms]) OR ("Reproductive Health"[Title/Abstract] OR "Health, Reproductive"[Title/Abstract])))) AND (((Refugees[MeSH Terms]) OR (Refugees[Title/Abstract] OR Refugee[Title/Abstract] OR "Political Asylum Seekers"[Title/Abstract] OR "Asylum Seeker, Political"[Title/Abstract] OR "Asylum Seekers, Political"[Title/Abstract] OR "Political Asylum Seeker"[Title/Abstract] OR "Seekers, Political Asylum"[Title/Abstract] OR "Political Refugees"[Title/Abstract] OR "Political Refugee"[Title/Abstract] OR "Refugee, Political"[Title/Abstract] OR "Refugees, Political"[Title/Abstract] OR "Asylum Seekers"[Title/Abstract] OR "Asylum Seeker"[Title/Abstract] OR "Seeker, Asylum"[Title/Abstract] OR "Seekers, Asylum"[Title/Abstract] OR "Displaced Persons"[Title/Abstract] OR "Displaced Person"[Title/Abstract] OR "Person, Displaced"[Title/Abstract] OR "Persons, Displaced"[Title/Abstract] OR "Internally Displaced Persons"[Title/Abstract] OR "Displaced Person, Internally"[Title/Abstract] OR "Displaced Persons, Internally"[Title/Abstract] OR "Internally Displaced Person"[Title/Abstract])) OR ((Transients and Migrants[MeSH Terms]) OR ("Transients and Migrants"[Title/Abstract] OR "Migrants and Transients"[Title/Abstract] OR Migrants[Title/Abstract] OR Migrant[Title/Abstract]))) | 124 | 05/01/2023 |
| BVS / BIREME  LILACS (8) | ((adolescent OR adolescents OR adolescence OR teens OR teen OR teenagers OR teenager OR youth OR youths OR "Adolescents, Female" OR "Adolescent, Female" OR "Female Adolescent" OR "Female Adolescents")) AND (("Health Vulnerability") OR ("Sexual Vulnerability") OR ("Sexual Health" OR "Health, Sexual") OR ("Reproductive Health" OR "Health, Reproductive")) AND ((refugees OR refugee OR "Political Asylum Seekers" OR "Asylum Seeker, Political" OR "Asylum Seekers, Political" OR "Political Asylum Seeker" OR "Seekers, Political Asylum" OR "Political Refugees" OR "Political Refugee" OR "Refugee, Political" OR "Refugees, Political" OR "Asylum Seekers" OR "Asylum Seeker" OR "Seeker, Asylum" OR "Seekers, Asylum" OR "Displaced Persons" OR "Displaced Person" OR "Person, Displaced" OR "Persons, Displaced" OR "Internally Displaced Persons" OR "Displaced Person, Internally" OR "Displaced Persons, Internally" OR "Internally Displaced Person") OR ("Transients and Migrants" OR "Migrants and Transients" OR migrants OR migrant)) AND ( db:("LILACS")) | 08 | 05/01/2023 |
| **SCOPUS** | ( ALL ( adolescent OR adolescents OR adolescence OR teens OR teen OR teenagers OR teenager OR youth OR youths OR "Adolescents, Female" OR "Adolescent, Female" OR "Female Adolescent" OR "Female Adolescents" ) ) AND ( ( TITLE-ABS-KEY ( "Health Vulnerability" ) OR TITLE-ABS-KEY ( "Sexual Vulnerability" ) OR TITLE-ABS-KEY ( "Sexual Health" OR "Health, Sexual" ) OR TITLE-ABS-KEY ( "Reproductive Health" OR "Health, Reproductive" ) ) ) AND ( ( TITLE-ABS-KEY ( refugees OR refugee OR "Political Asylum Seekers" OR "Asylum Seeker, Political" OR "Asylum Seekers, Political" OR "Political Asylum Seeker" OR "Seekers, Political Asylum" OR "Political Refugees" OR "Political Refugee" OR "Refugee, Political" OR "Refugees, Political" OR "Asylum Seekers" OR "Asylum Seeker" OR "Seeker, Asylum" OR "Seekers, Asylum" OR "Displaced Persons" OR "Displaced Person" OR "Person, Displaced" OR "Persons, Displaced" OR "Internally Displaced Persons" OR "Displaced Person, Internally" OR "Displaced Persons, Internally" OR "Internally Displaced Person" ) OR TITLE-ABS-KEY ( "Transients and Migrants" OR "Migrants and Transients" OR migrants OR migrant ) ) ) | 692 | 05/01/2023 |
| **WEB OF SCIENCE** | Adolescent OR Adolescents OR Adolescence OR Teens OR Teen OR Teenagers OR Teenager OR Youth OR Youths OR "Adolescents, Female" OR "Adolescent, Female" OR "Female Adolescent" OR "Female Adolescents" (Tópico) AND "Health Vulnerability" (Tópico) or "Sexual Vulnerability" (Tópico) or "Sexual Health" OR "Health, Sexual" (Tópico) or "Reproductive Health" OR "Health, Reproductive" (Tópico) AND Refugees OR Refugee OR "Political Asylum Seekers" OR "Asylum Seeker, Political" OR "Asylum Seekers, Political" OR "Political Asylum Seeker" OR "Seekers, Political Asylum" OR "Political Refugees" OR "Political Refugee" OR "Refugee, Political" OR "Refugees, Political" OR "Asylum Seekers" OR "Asylum Seeker" OR "Seeker, Asylum" OR "Seekers, Asylum" OR "Displaced Persons" OR "Displaced Person" OR "Person, Displaced" OR "Persons, Displaced" OR "Internally Displaced Persons" OR "Displaced Person, Internally" OR "Displaced Persons, Internally" OR "Internally Displaced Person" (Tópico) or "Transients and Migrants" OR "Migrants and Transients" OR Migrants OR Migrant (Tópico)  LINK PERMANENTE: <https://www.webofscience.com/wos/alldb/summary/8c8d19d0-d7c6-4f8a-a2ed-12cecf15f582-68e4ee3e/relevance/1> | 763 | 05/01/2023 |
| **EMBASE** | (adolescent OR adolescents OR adolescence OR teens OR teen OR teenagers OR teenager OR youth OR youths OR 'adolescents, female' OR 'adolescent, female' OR 'female adolescent' OR 'female adolescents') AND ('health vulnerability' OR 'sexual vulnerability' OR 'sexual health'/syn OR 'reproductive health'/syn) AND ('refugee'/syn OR 'migrant'/syn) AND ([embase]/lim NOT ([embase]/lim AND [medline]/lim) OR ([medline]/lim NOT ([embase]/lim AND [medline]/lim) NOT ([embase classic]/lim AND [medline]/lim))) | 255 | 05/01/2023 |
| **TOTAL** |  | 2.173 |  |
| **TOTAL DE REFERÊNCIAS EM DUPLICIDADE** | **853 ARTIGOS EXCLUÍDOS POR DUPLICIDADES NO ENDNOTE WEB**  **030 ARTIGOS EXCLUÍDOS POR DUPLICIDADES NO RAYYAN** | 883 |  |
| **TOTAL APÓS EXCLUSÃO DE DUPLICIDADE** |  | 1.290 |  |


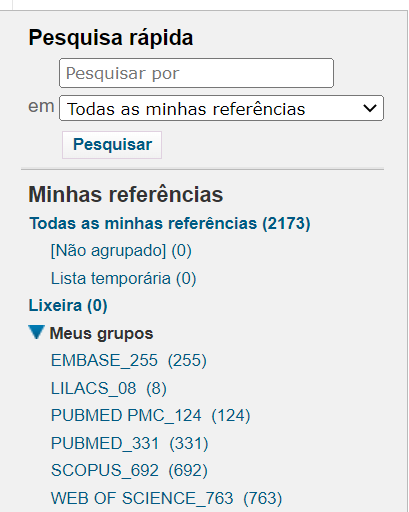

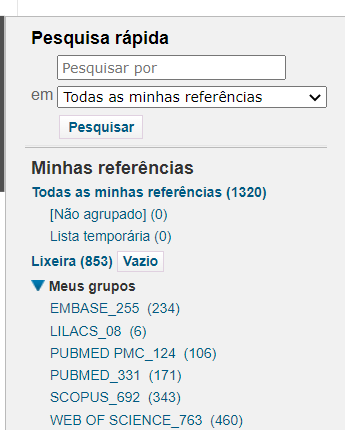


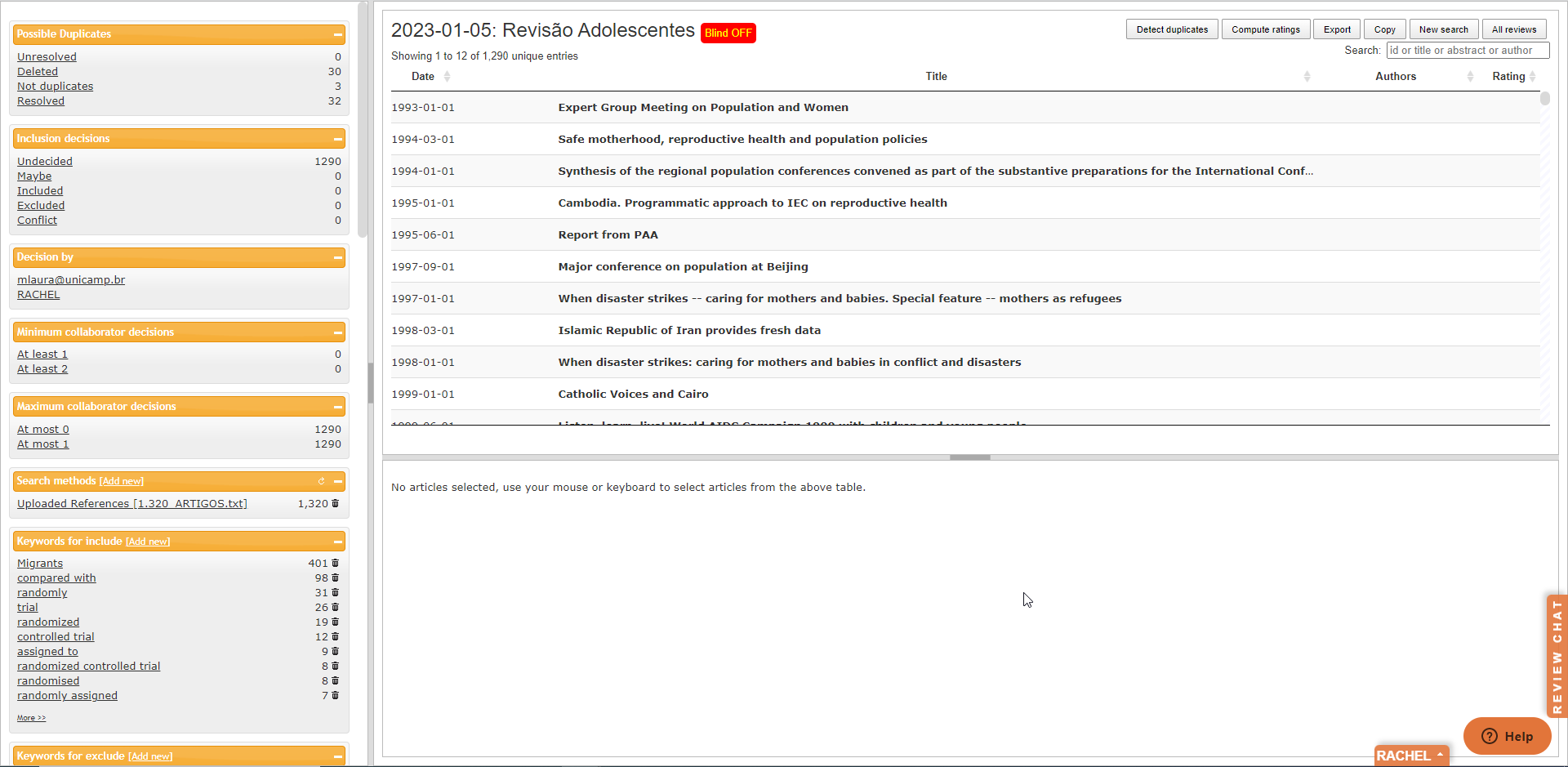


24/11/2022

## History and Search Details

Parte superior do formulário

Download

Parte inferior do formulário

Delete

| **Search** | **Actions** | **Details** | **Query** | **Results** | **Time** |
| --- | --- | --- | --- | --- | --- |
| #26 |  |  | Search: **(((((Women OR Girls OR Girl OR Woman OR "Women's Groups" OR "Women Groups" OR "Women's Group") OR (Female OR Females)) AND ((("Health Vulnerability") OR ("Sexual Vulnerability")) OR (Vulnerability))) AND (((Sexual Health[MeSH Terms]) OR ("Sexual Health"[Title/Abstract] OR "Health, Sexual"[Title/Abstract])) OR ((Reproductive Health[MeSH Terms]) OR ("Reproductive Health"[Title/Abstract] OR "Health, Reproductive"[Title/Abstract])))) AND (((((Coronavirus Infections[MeSH Terms]) OR ("Coronavirus Infections"[Title/Abstract] OR "Coronavirus Infection"[Title/Abstract] OR "Infection, Coronavirus"[Title/Abstract] OR "Infections, Coronavirus"[Title/Abstract] OR "Middle East Respiratory Syndrome"[Title/Abstract] OR "MERS (Middle East Respiratory Syndrome)"[Title/Abstract])) OR ((COVID-19[MeSH Terms]) OR ("COVID-19"[Title/Abstract] OR "COVID 19"[Title/Abstract] OR "COVID-19 Virus Disease"[Title/Abstract] OR "COVID 19 Virus Disease"[Title/Abstract] OR "COVID-19 Virus Diseases"[Title/Abstract] OR "Disease, COVID-19 Virus"[Title/Abstract] OR "Virus Disease, COVID-19"[Title/Abstract] OR "COVID-19 Virus Infection"[Title/Abstract] OR "COVID 19 Virus Infection"[Title/Abstract] OR "COVID-19 Virus Infections"[Title/Abstract] OR "Infection, COVID-19 Virus"[Title/Abstract] OR "Virus Infection, COVID-19"[Title/Abstract] OR "2019-nCoV Infection"[Title/Abstract] OR "2019 nCoV Infection"[Title/Abstract] OR "2019-nCoV Infections"[Title/Abstract] OR "Infection, 2019-nCoV"[Title/Abstract] OR "Coronavirus Disease-19"[Title/Abstract] OR "Coronavirus Disease 19"[Title/Abstract] OR "2019 Novel Coronavirus Disease"[Title/Abstract] OR "2019 Novel Coronavirus Infection"[Title/Abstract] OR "2019-nCoV Disease"[Title/Abstract] OR "2019 nCoV Disease"[Title/Abstract] OR "2019-nCoV Diseases"[Title/Abstract] OR "Disease, 2019-nCoV"[Title/Abstract] OR COVID19[Title/Abstract] OR "Coronavirus Disease 2019"[Title/Abstract] OR "Disease 2019, Coronavirus"[Title/Abstract] OR "SARS Coronavirus 2 Infection"[Title/Abstract] OR "SARS-CoV-2 Infection"[Title/Abstract] OR "Infection, SARS-CoV-2"[Title/Abstract] OR "SARS CoV 2 Infection"[Title/Abstract] OR "SARS-CoV-2 Infections"[Title/Abstract] OR "COVID-19 Pandemic"[Title/Abstract] OR "COVID 19 Pandemic"[Title/Abstract] OR "COVID-19 Pandemics"[Title/Abstract] OR "Pandemic, COVID-19"[Title/Abstract]))) OR ("2019 novel coronavirus disease" OR "2019 novel coronavirus epidemic" OR "2019 novel coronavirus infection" OR "2019-nCoV disease" OR "2019-nCoV infection" OR "coronavirus disease 2" OR "coronavirus disease 2010" OR "coronavirus disease 2019 pneumonia" OR "coronavirus disease-19" OR "coronavirus infection 2019" OR "COVID" OR "COVID 19" OR "COVID 19 induced pneumonia" OR "COVID 2019" OR "COVID-10" OR "COVID-19" OR "COVID-19 induced pneumonia" OR "COVID-19 pneumonia" OR "COVID19" OR "nCoV 2019 disease" OR "nCoV 2019 infection" OR "new coronavirus pneumonia" OR "novel coronavirus 2019 disease" OR "novel coronavirus 2019 infection" OR "novel coronavirus disease 2019" OR "novel coronavirus infected pneumonia" OR "novel coronavirus infection 2019" OR "novel coronavirus pneumonia" OR "paucisymptomatic coronavirus disease 2019" OR "SARS coronavirus 2 infection" OR "SARS coronavirus 2 pneumonia" OR "SARS-CoV-2 disease" OR "SARS-CoV-2 infection" OR "SARS-CoV-2 pneumonia" OR "SARS-CoV2 disease" OR "SARS-CoV2 infection" OR "SARSCoV2 disease" OR "SARSCoV2 infection" OR "severe acute respiratory syndrome 2" OR "severe acute respiratory syndrome 2 pneumonia" OR "severe acute respiratory syndrome coronavirus 2 infection" OR "severe acute respiratory syndrome coronavirus 2019 infection" OR "severe acute respiratory syndrome CoV-2 infection" OR "Wuhan coronavirus disease" OR "Wuhan coronavirus infection")) OR ((SARS-CoV-2[MeSH Terms]) OR ("SARS-CoV-2"[Title/Abstract] OR "Coronavirus Disease 2019 Virus"[Title/Abstract] OR "2019 Novel Coronavirus"[Title/Abstract] OR "2019 Novel Coronaviruses"[Title/Abstract] OR "Coronavirus, 2019 Novel"[Title/Abstract] OR "Novel Coronavirus, 2019"[Title/Abstract] OR "Wuhan Seafood Market Pneumonia Virus"[Title/Abstract] OR "SARS-CoV-2 Virus"[Title/Abstract] OR "SARS CoV 2 Virus"[Title/Abstract] OR "SARS-CoV-2 Viruses"[Title/Abstract] OR "Virus, SARS-CoV-2"[Title/Abstract] OR "2019-nCoV"[Title/Abstract] OR "COVID-19 Virus"[Title/Abstract] OR "COVID 19 Virus"[Title/Abstract] OR "COVID-19 Viruses"[Title/Abstract] OR "Virus, COVID-19"[Title/Abstract] OR "Wuhan Coronavirus"[Title/Abstract] OR "Coronavirus, Wuhan"[Title/Abstract] OR "SARS Coronavirus 2"[Title/Abstract] OR "Coronavirus 2, SARS"[Title/Abstract] OR "Severe Acute Respiratory Syndrome Coronavirus 2"[Title/Abstract])))) AND (((Pregnant Women[MeSH Terms]) OR ("Pregnant Women"[Title/Abstract] OR "Pregnant Woman"[Title/Abstract] OR "Woman, Pregnant"[Title/Abstract] OR "Women, Pregnant"[Title/Abstract])) OR ((Pregnancy[MeSH Terms]) OR (Pregnancy[Title/Abstract] OR Pregnancies[Title/Abstract] OR Gestation[Title/Abstract])))** | [24](https://pubmed.ncbi.nlm.nih.gov/?term=longquery643d3fe640d80042be53&format=abstract&size=200&sort=relevance) | 11:36:55 |
| #25 |  |  | Search: **((Pregnant Women[MeSH Terms]) OR ("Pregnant Women"[Title/Abstract] OR "Pregnant Woman"[Title/Abstract] OR "Woman, Pregnant"[Title/Abstract] OR "Women, Pregnant"[Title/Abstract])) OR ((Pregnancy[MeSH Terms]) OR (Pregnancy[Title/Abstract] OR Pregnancies[Title/Abstract] OR Gestation[Title/Abstract]))** | [1,124,624](https://pubmed.ncbi.nlm.nih.gov/?term=%28%28Pregnant+Women%5BMeSH+Terms%5D%29+OR+%28%22Pregnant+Women%22%5BTitle%2FAbstract%5D+OR+%22Pregnant+Woman%22%5BTitle%2FAbstract%5D+OR+%22Woman%2C+Pregnant%22%5BTitle%2FAbstract%5D+OR+%22Women%2C+Pregnant%22%5BTitle%2FAbstract%5D%29%29+OR+%28%28Pregnancy%5BMeSH+Terms%5D%29+OR+%28Pregnancy%5BTitle%2FAbstract%5D+OR+Pregnancies%5BTitle%2FAbstract%5D+OR+Gestation%5BTitle%2FAbstract%5D%29%29&size=200&ac=no&sort=relevance) | 11:35:27 |
| #24 |  |  | Search: **(Pregnancy[MeSH Terms]) OR (Pregnancy[Title/Abstract] OR Pregnancies[Title/Abstract] OR Gestation[Title/Abstract])** | [1,114,040](https://pubmed.ncbi.nlm.nih.gov/?term=%28Pregnancy%5BMeSH+Terms%5D%29+OR+%28Pregnancy%5BTitle%2FAbstract%5D+OR+Pregnancies%5BTitle%2FAbstract%5D+OR+Gestation%5BTitle%2FAbstract%5D%29&size=200&ac=no&sort=relevance) | 11:35:20 |
| #23 |  |  | Search: **(Pregnant Women[MeSH Terms]) OR ("Pregnant Women"[Title/Abstract] OR "Pregnant Woman"[Title/Abstract] OR "Woman, Pregnant"[Title/Abstract] OR "Women, Pregnant"[Title/Abstract])** | [125,006](https://pubmed.ncbi.nlm.nih.gov/?term=%28Pregnant+Women%5BMeSH+Terms%5D%29+OR+%28%22Pregnant+Women%22%5BTitle%2FAbstract%5D+OR+%22Pregnant+Woman%22%5BTitle%2FAbstract%5D+OR+%22Woman%2C+Pregnant%22%5BTitle%2FAbstract%5D+OR+%22Women%2C+Pregnant%22%5BTitle%2FAbstract%5D%29&size=200&ac=no&sort=relevance) | 11:34:57 |
| #22 |  |  | Search: **((((Women OR Girls OR Girl OR Woman OR "Women's Groups" OR "Women Groups" OR "Women's Group") OR (Female OR Females)) AND ((("Health Vulnerability") OR ("Sexual Vulnerability")) OR (Vulnerability))) AND (((Sexual Health[MeSH Terms]) OR ("Sexual Health"[Title/Abstract] OR "Health, Sexual"[Title/Abstract])) OR ((Reproductive Health[MeSH Terms]) OR ("Reproductive Health"[Title/Abstract] OR "Health, Reproductive"[Title/Abstract])))) AND (((((Coronavirus Infections[MeSH Terms]) OR ("Coronavirus Infections"[Title/Abstract] OR "Coronavirus Infection"[Title/Abstract] OR "Infection, Coronavirus"[Title/Abstract] OR "Infections, Coronavirus"[Title/Abstract] OR "Middle East Respiratory Syndrome"[Title/Abstract] OR "MERS (Middle East Respiratory Syndrome)"[Title/Abstract])) OR ((COVID-19[MeSH Terms]) OR ("COVID-19"[Title/Abstract] OR "COVID 19"[Title/Abstract] OR "COVID-19 Virus Disease"[Title/Abstract] OR "COVID 19 Virus Disease"[Title/Abstract] OR "COVID-19 Virus Diseases"[Title/Abstract] OR "Disease, COVID-19 Virus"[Title/Abstract] OR "Virus Disease, COVID-19"[Title/Abstract] OR "COVID-19 Virus Infection"[Title/Abstract] OR "COVID 19 Virus Infection"[Title/Abstract] OR "COVID-19 Virus Infections"[Title/Abstract] OR "Infection, COVID-19 Virus"[Title/Abstract] OR "Virus Infection, COVID-19"[Title/Abstract] OR "2019-nCoV Infection"[Title/Abstract] OR "2019 nCoV Infection"[Title/Abstract] OR "2019-nCoV Infections"[Title/Abstract] OR "Infection, 2019-nCoV"[Title/Abstract] OR "Coronavirus Disease-19"[Title/Abstract] OR "Coronavirus Disease 19"[Title/Abstract] OR "2019 Novel Coronavirus Disease"[Title/Abstract] OR "2019 Novel Coronavirus Infection"[Title/Abstract] OR "2019-nCoV Disease"[Title/Abstract] OR "2019 nCoV Disease"[Title/Abstract] OR "2019-nCoV Diseases"[Title/Abstract] OR "Disease, 2019-nCoV"[Title/Abstract] OR COVID19[Title/Abstract] OR "Coronavirus Disease 2019"[Title/Abstract] OR "Disease 2019, Coronavirus"[Title/Abstract] OR "SARS Coronavirus 2 Infection"[Title/Abstract] OR "SARS-CoV-2 Infection"[Title/Abstract] OR "Infection, SARS-CoV-2"[Title/Abstract] OR "SARS CoV 2 Infection"[Title/Abstract] OR "SARS-CoV-2 Infections"[Title/Abstract] OR "COVID-19 Pandemic"[Title/Abstract] OR "COVID 19 Pandemic"[Title/Abstract] OR "COVID-19 Pandemics"[Title/Abstract] OR "Pandemic, COVID-19"[Title/Abstract]))) OR ("2019 novel coronavirus disease" OR "2019 novel coronavirus epidemic" OR "2019 novel coronavirus infection" OR "2019-nCoV disease" OR "2019-nCoV infection" OR "coronavirus disease 2" OR "coronavirus disease 2010" OR "coronavirus disease 2019 pneumonia" OR "coronavirus disease-19" OR "coronavirus infection 2019" OR "COVID" OR "COVID 19" OR "COVID 19 induced pneumonia" OR "COVID 2019" OR "COVID-10" OR "COVID-19" OR "COVID-19 induced pneumonia" OR "COVID-19 pneumonia" OR "COVID19" OR "nCoV 2019 disease" OR "nCoV 2019 infection" OR "new coronavirus pneumonia" OR "novel coronavirus 2019 disease" OR "novel coronavirus 2019 infection" OR "novel coronavirus disease 2019" OR "novel coronavirus infected pneumonia" OR "novel coronavirus infection 2019" OR "novel coronavirus pneumonia" OR "paucisymptomatic coronavirus disease 2019" OR "SARS coronavirus 2 infection" OR "SARS coronavirus 2 pneumonia" OR "SARS-CoV-2 disease" OR "SARS-CoV-2 infection" OR "SARS-CoV-2 pneumonia" OR "SARS-CoV2 disease" OR "SARS-CoV2 infection" OR "SARSCoV2 disease" OR "SARSCoV2 infection" OR "severe acute respiratory syndrome 2" OR "severe acute respiratory syndrome 2 pneumonia" OR "severe acute respiratory syndrome coronavirus 2 infection" OR "severe acute respiratory syndrome coronavirus 2019 infection" OR "severe acute respiratory syndrome CoV-2 infection" OR "Wuhan coronavirus disease" OR "Wuhan coronavirus infection")) OR ((SARS-CoV-2[MeSH Terms]) OR ("SARS-CoV-2"[Title/Abstract] OR "Coronavirus Disease 2019 Virus"[Title/Abstract] OR "2019 Novel Coronavirus"[Title/Abstract] OR "2019 Novel Coronaviruses"[Title/Abstract] OR "Coronavirus, 2019 Novel"[Title/Abstract] OR "Novel Coronavirus, 2019"[Title/Abstract] OR "Wuhan Seafood Market Pneumonia Virus"[Title/Abstract] OR "SARS-CoV-2 Virus"[Title/Abstract] OR "SARS CoV 2 Virus"[Title/Abstract] OR "SARS-CoV-2 Viruses"[Title/Abstract] OR "Virus, SARS-CoV-2"[Title/Abstract] OR "2019-nCoV"[Title/Abstract] OR "COVID-19 Virus"[Title/Abstract] OR "COVID 19 Virus"[Title/Abstract] OR "COVID-19 Viruses"[Title/Abstract] OR "Virus, COVID-19"[Title/Abstract] OR "Wuhan Coronavirus"[Title/Abstract] OR "Coronavirus, Wuhan"[Title/Abstract] OR "SARS Coronavirus 2"[Title/Abstract] OR "Coronavirus 2, SARS"[Title/Abstract] OR "Severe Acute Respiratory Syndrome Coronavirus 2"[Title/Abstract])))** | [45](https://pubmed.ncbi.nlm.nih.gov/?term=longqueryaa6f58099d7c70691ed9&size=200&ac=no&sort=relevance) | 11:30:33 |
| #21 |  |  | Search: **((((Coronavirus Infections[MeSH Terms]) OR ("Coronavirus Infections"[Title/Abstract] OR "Coronavirus Infection"[Title/Abstract] OR "Infection, Coronavirus"[Title/Abstract] OR "Infections, Coronavirus"[Title/Abstract] OR "Middle East Respiratory Syndrome"[Title/Abstract] OR "MERS (Middle East Respiratory Syndrome)"[Title/Abstract])) OR ((COVID-19[MeSH Terms]) OR ("COVID-19"[Title/Abstract] OR "COVID 19"[Title/Abstract] OR "COVID-19 Virus Disease"[Title/Abstract] OR "COVID 19 Virus Disease"[Title/Abstract] OR "COVID-19 Virus Diseases"[Title/Abstract] OR "Disease, COVID-19 Virus"[Title/Abstract] OR "Virus Disease, COVID-19"[Title/Abstract] OR "COVID-19 Virus Infection"[Title/Abstract] OR "COVID 19 Virus Infection"[Title/Abstract] OR "COVID-19 Virus Infections"[Title/Abstract] OR "Infection, COVID-19 Virus"[Title/Abstract] OR "Virus Infection, COVID-19"[Title/Abstract] OR "2019-nCoV Infection"[Title/Abstract] OR "2019 nCoV Infection"[Title/Abstract] OR "2019-nCoV Infections"[Title/Abstract] OR "Infection, 2019-nCoV"[Title/Abstract] OR "Coronavirus Disease-19"[Title/Abstract] OR "Coronavirus Disease 19"[Title/Abstract] OR "2019 Novel Coronavirus Disease"[Title/Abstract] OR "2019 Novel Coronavirus Infection"[Title/Abstract] OR "2019-nCoV Disease"[Title/Abstract] OR "2019 nCoV Disease"[Title/Abstract] OR "2019-nCoV Diseases"[Title/Abstract] OR "Disease, 2019-nCoV"[Title/Abstract] OR COVID19[Title/Abstract] OR "Coronavirus Disease 2019"[Title/Abstract] OR "Disease 2019, Coronavirus"[Title/Abstract] OR "SARS Coronavirus 2 Infection"[Title/Abstract] OR "SARS-CoV-2 Infection"[Title/Abstract] OR "Infection, SARS-CoV-2"[Title/Abstract] OR "SARS CoV 2 Infection"[Title/Abstract] OR "SARS-CoV-2 Infections"[Title/Abstract] OR "COVID-19 Pandemic"[Title/Abstract] OR "COVID 19 Pandemic"[Title/Abstract] OR "COVID-19 Pandemics"[Title/Abstract] OR "Pandemic, COVID-19"[Title/Abstract]))) OR ("2019 novel coronavirus disease" OR "2019 novel coronavirus epidemic" OR "2019 novel coronavirus infection" OR "2019-nCoV disease" OR "2019-nCoV infection" OR "coronavirus disease 2" OR "coronavirus disease 2010" OR "coronavirus disease 2019 pneumonia" OR "coronavirus disease-19" OR "coronavirus infection 2019" OR "COVID" OR "COVID 19" OR "COVID 19 induced pneumonia" OR "COVID 2019" OR "COVID-10" OR "COVID-19" OR "COVID-19 induced pneumonia" OR "COVID-19 pneumonia" OR "COVID19" OR "nCoV 2019 disease" OR "nCoV 2019 infection" OR "new coronavirus pneumonia" OR "novel coronavirus 2019 disease" OR "novel coronavirus 2019 infection" OR "novel coronavirus disease 2019" OR "novel coronavirus infected pneumonia" OR "novel coronavirus infection 2019" OR "novel coronavirus pneumonia" OR "paucisymptomatic coronavirus disease 2019" OR "SARS coronavirus 2 infection" OR "SARS coronavirus 2 pneumonia" OR "SARS-CoV-2 disease" OR "SARS-CoV-2 infection" OR "SARS-CoV-2 pneumonia" OR "SARS-CoV2 disease" OR "SARS-CoV2 infection" OR "SARSCoV2 disease" OR "SARSCoV2 infection" OR "severe acute respiratory syndrome 2" OR "severe acute respiratory syndrome 2 pneumonia" OR "severe acute respiratory syndrome coronavirus 2 infection" OR "severe acute respiratory syndrome coronavirus 2019 infection" OR "severe acute respiratory syndrome CoV-2 infection" OR "Wuhan coronavirus disease" OR "Wuhan coronavirus infection")) OR ((SARS-CoV-2[MeSH Terms]) OR ("SARS-CoV-2"[Title/Abstract] OR "Coronavirus Disease 2019 Virus"[Title/Abstract] OR "2019 Novel Coronavirus"[Title/Abstract] OR "2019 Novel Coronaviruses"[Title/Abstract] OR "Coronavirus, 2019 Novel"[Title/Abstract] OR "Novel Coronavirus, 2019"[Title/Abstract] OR "Wuhan Seafood Market Pneumonia Virus"[Title/Abstract] OR "SARS-CoV-2 Virus"[Title/Abstract] OR "SARS CoV 2 Virus"[Title/Abstract] OR "SARS-CoV-2 Viruses"[Title/Abstract] OR "Virus, SARS-CoV-2"[Title/Abstract] OR "2019-nCoV"[Title/Abstract] OR "COVID-19 Virus"[Title/Abstract] OR "COVID 19 Virus"[Title/Abstract] OR "COVID-19 Viruses"[Title/Abstract] OR "Virus, COVID-19"[Title/Abstract] OR "Wuhan Coronavirus"[Title/Abstract] OR "Coronavirus, Wuhan"[Title/Abstract] OR "SARS Coronavirus 2"[Title/Abstract] OR "Coronavirus 2, SARS"[Title/Abstract] OR "Severe Acute Respiratory Syndrome Coronavirus 2"[Title/Abstract]))** | [325,501](https://pubmed.ncbi.nlm.nih.gov/?term=longqueryc338f505c876a28adec1&size=200&ac=no&sort=relevance) | 11:29:56 |
| #20 |  |  | Search: **(SARS-CoV-2[MeSH Terms]) OR ("SARS-CoV-2"[Title/Abstract] OR "Coronavirus Disease 2019 Virus"[Title/Abstract] OR "2019 Novel Coronavirus"[Title/Abstract] OR "2019 Novel Coronaviruses"[Title/Abstract] OR "Coronavirus, 2019 Novel"[Title/Abstract] OR "Novel Coronavirus, 2019"[Title/Abstract] OR "Wuhan Seafood Market Pneumonia Virus"[Title/Abstract] OR "SARS-CoV-2 Virus"[Title/Abstract] OR "SARS CoV 2 Virus"[Title/Abstract] OR "SARS-CoV-2 Viruses"[Title/Abstract] OR "Virus, SARS-CoV-2"[Title/Abstract] OR "2019-nCoV"[Title/Abstract] OR "COVID-19 Virus"[Title/Abstract] OR "COVID 19 Virus"[Title/Abstract] OR "COVID-19 Viruses"[Title/Abstract] OR "Virus, COVID-19"[Title/Abstract] OR "Wuhan Coronavirus"[Title/Abstract] OR "Coronavirus, Wuhan"[Title/Abstract] OR "SARS Coronavirus 2"[Title/Abstract] OR "Coronavirus 2, SARS"[Title/Abstract] OR "Severe Acute Respiratory Syndrome Coronavirus 2"[Title/Abstract])** | [187,711](https://pubmed.ncbi.nlm.nih.gov/?term=%28SARS-CoV-2%5BMeSH+Terms%5D%29+OR+%28%22SARS-CoV-2%22%5BTitle%2FAbstract%5D+OR+%22Coronavirus+Disease+2019+Virus%22%5BTitle%2FAbstract%5D+OR+%222019+Novel+Coronavirus%22%5BTitle%2FAbstract%5D+OR+%222019+Novel+Coronaviruses%22%5BTitle%2FAbstract%5D+OR+%22Coronavirus%2C+2019+Novel%22%5BTitle%2FAbstract%5D+OR+%22Novel+Coronavirus%2C+2019%22%5BTitle%2FAbstract%5D+OR+%22Wuhan+Seafood+Market+Pneumonia+Virus%22%5BTitle%2FAbstract%5D+OR+%22SARS-CoV-2+Virus%22%5BTitle%2FAbstract%5D+OR+%22SARS+CoV+2+Virus%22%5BTitle%2FAbstract%5D+OR+%22SARS-CoV-2+Viruses%22%5BTitle%2FAbstract%5D+OR+%22Virus%2C+SARS-CoV-2%22%5BTitle%2FAbstract%5D+OR+%222019-nCoV%22%5BTitle%2FAbstract%5D+OR+%22COVID-19+Virus%22%5BTitle%2FAbstract%5D+OR+%22COVID+19+Virus%22%5BTitle%2FAbstract%5D+OR+%22COVID-19+Viruses%22%5BTitle%2FAbstract%5D+OR+%22Virus%2C+COVID-19%22%5BTitle%2FAbstract%5D+OR+%22Wuhan+Coronavirus%22%5BTitle%2FAbstract%5D+OR+%22Coronavirus%2C+Wuhan%22%5BTitle%2FAbstract%5D+OR+%22SARS+Coronavirus+2%22%5BTitle%2FAbstract%5D+OR+%22Coronavirus+2%2C+SARS%22%5BTitle%2FAbstract%5D+OR+%22Severe+Acute+Respiratory+Syndrome+Coronavirus+2%22%5BTitle%2FAbstract%5D%29&size=200&ac=no&sort=relevance) | 11:29:37 |
| #19 |  |  | Search: **"2019 novel coronavirus disease" OR "2019 novel coronavirus epidemic" OR "2019 novel coronavirus infection" OR "2019-nCoV disease" OR "2019-nCoV infection" OR "coronavirus disease 2" OR "coronavirus disease 2010" OR "coronavirus disease 2019 pneumonia" OR "coronavirus disease-19" OR "coronavirus infection 2019" OR "COVID" OR "COVID 19" OR "COVID 19 induced pneumonia" OR "COVID 2019" OR "COVID-10" OR "COVID-19" OR "COVID-19 induced pneumonia" OR "COVID-19 pneumonia" OR "COVID19" OR "nCoV 2019 disease" OR "nCoV 2019 infection" OR "new coronavirus pneumonia" OR "novel coronavirus 2019 disease" OR "novel coronavirus 2019 infection" OR "novel coronavirus disease 2019" OR "novel coronavirus infected pneumonia" OR "novel coronavirus infection 2019" OR "novel coronavirus pneumonia" OR "paucisymptomatic coronavirus disease 2019" OR "SARS coronavirus 2 infection" OR "SARS coronavirus 2 pneumonia" OR "SARS-CoV-2 disease" OR "SARS-CoV-2 infection" OR "SARS-CoV-2 pneumonia" OR "SARS-CoV2 disease" OR "SARS-CoV2 infection" OR "SARSCoV2 disease" OR "SARSCoV2 infection" OR "severe acute respiratory syndrome 2" OR "severe acute respiratory syndrome 2 pneumonia" OR "severe acute respiratory syndrome coronavirus 2 infection" OR "severe acute respiratory syndrome coronavirus 2019 infection" OR "severe acute respiratory syndrome CoV-2 infection" OR "Wuhan coronavirus disease" OR "Wuhan coronavirus infection"** | [310,550](https://pubmed.ncbi.nlm.nih.gov/?term=%222019+novel+coronavirus+disease%22+OR+%222019+novel+coronavirus+epidemic%22+OR+%222019+novel+coronavirus+infection%22+OR+%222019-nCoV+disease%22+OR+%222019-nCoV+infection%22+OR+%22coronavirus+disease+2%22+OR+%22coronavirus+disease+2010%22+OR+%22coronavirus+disease+2019+pneumonia%22+OR+%22coronavirus+disease-19%22+OR+%22coronavirus+infection+2019%22+OR+%22COVID%22+OR+%22COVID+19%22+OR+%22COVID+19+induced+pneumonia%22+OR+%22COVID+2019%22+OR+%22COVID-10%22+OR+%22COVID-19%22+OR+%22COVID-19+induced+pneumonia%22+OR+%22COVID-19+pneumonia%22+OR+%22COVID19%22+OR+%22nCoV+2019+disease%22+OR+%22nCoV+2019+infection%22+OR+%22new+coronavirus+pneumonia%22+OR+%22novel+coronavirus+2019+disease%22+OR+%22novel+coronavirus+2019+infection%22+OR+%22novel+coronavirus+disease+2019%22+OR+%22novel+coronavirus+infected+pneumonia%22+OR+%22novel+coronavirus+infection+2019%22+OR+%22novel+coronavirus+pneumonia%22+OR+%22paucisymptomatic+coronavirus+disease+2019%22+OR+%22SARS+coronavirus+2+infection%22+OR+%22SARS+coronavirus+2+pneumonia%22+OR+%22SARS-CoV-2+disease%22+OR+%22SARS-CoV-2+infection%22+OR+%22SARS-CoV-2+pneumonia%22+OR+%22SARS-CoV2+disease%22+OR+%22SARS-CoV2+infection%22+OR+%22SARSCoV2+disease%22+OR+%22SARSCoV2+infection%22+OR+%22severe+acute+respiratory+syndrome+2%22+OR+%22severe+acute+respiratory+syndrome+2+pneumonia%22+OR+%22severe+acute+respiratory+syndrome+coronavirus+2+infection%22+OR+%22severe+acute+respiratory+syndrome+coronavirus+2019+infection%22+OR+%22severe+acute+respiratory+syndrome+CoV-2+infection%22+OR+%22Wuhan+coronavirus+disease%22+OR+%22Wuhan+coronavirus+infection%22&size=200&ac=no&sort=relevance) | 11:29:12 |
| #18 |  |  | Search: **(COVID-19[MeSH Terms]) OR ("COVID-19"[Title/Abstract] OR "COVID 19"[Title/Abstract] OR "COVID-19 Virus Disease"[Title/Abstract] OR "COVID 19 Virus Disease"[Title/Abstract] OR "COVID-19 Virus Diseases"[Title/Abstract] OR "Disease, COVID-19 Virus"[Title/Abstract] OR "Virus Disease, COVID-19"[Title/Abstract] OR "COVID-19 Virus Infection"[Title/Abstract] OR "COVID 19 Virus Infection"[Title/Abstract] OR "COVID-19 Virus Infections"[Title/Abstract] OR "Infection, COVID-19 Virus"[Title/Abstract] OR "Virus Infection, COVID-19"[Title/Abstract] OR "2019-nCoV Infection"[Title/Abstract] OR "2019 nCoV Infection"[Title/Abstract] OR "2019-nCoV Infections"[Title/Abstract] OR "Infection, 2019-nCoV"[Title/Abstract] OR "Coronavirus Disease-19"[Title/Abstract] OR "Coronavirus Disease 19"[Title/Abstract] OR "2019 Novel Coronavirus Disease"[Title/Abstract] OR "2019 Novel Coronavirus Infection"[Title/Abstract] OR "2019-nCoV Disease"[Title/Abstract] OR "2019 nCoV Disease"[Title/Abstract] OR "2019-nCoV Diseases"[Title/Abstract] OR "Disease, 2019-nCoV"[Title/Abstract] OR COVID19[Title/Abstract] OR "Coronavirus Disease 2019"[Title/Abstract] OR "Disease 2019, Coronavirus"[Title/Abstract] OR "SARS Coronavirus 2 Infection"[Title/Abstract] OR "SARS-CoV-2 Infection"[Title/Abstract] OR "Infection, SARS-CoV-2"[Title/Abstract] OR "SARS CoV 2 Infection"[Title/Abstract] OR "SARS-CoV-2 Infections"[Title/Abstract] OR "COVID-19 Pandemic"[Title/Abstract] OR "COVID 19 Pandemic"[Title/Abstract] OR "COVID-19 Pandemics"[Title/Abstract] OR "Pandemic, COVID-19"[Title/Abstract])** | [302,838](https://pubmed.ncbi.nlm.nih.gov/?term=%28COVID-19%5BMeSH+Terms%5D%29+OR+%28%22COVID-19%22%5BTitle%2FAbstract%5D+OR+%22COVID+19%22%5BTitle%2FAbstract%5D+OR+%22COVID-19+Virus+Disease%22%5BTitle%2FAbstract%5D+OR+%22COVID+19+Virus+Disease%22%5BTitle%2FAbstract%5D+OR+%22COVID-19+Virus+Diseases%22%5BTitle%2FAbstract%5D+OR+%22Disease%2C+COVID-19+Virus%22%5BTitle%2FAbstract%5D+OR+%22Virus+Disease%2C+COVID-19%22%5BTitle%2FAbstract%5D+OR+%22COVID-19+Virus+Infection%22%5BTitle%2FAbstract%5D+OR+%22COVID+19+Virus+Infection%22%5BTitle%2FAbstract%5D+OR+%22COVID-19+Virus+Infections%22%5BTitle%2FAbstract%5D+OR+%22Infection%2C+COVID-19+Virus%22%5BTitle%2FAbstract%5D+OR+%22Virus+Infection%2C+COVID-19%22%5BTitle%2FAbstract%5D+OR+%222019-nCoV+Infection%22%5BTitle%2FAbstract%5D+OR+%222019+nCoV+Infection%22%5BTitle%2FAbstract%5D+OR+%222019-nCoV+Infections%22%5BTitle%2FAbstract%5D+OR+%22Infection%2C+2019-nCoV%22%5BTitle%2FAbstract%5D+OR+%22Coronavirus+Disease-19%22%5BTitle%2FAbstract%5D+OR+%22Coronavirus+Disease+19%22%5BTitle%2FAbstract%5D+OR+%222019+Novel+Coronavirus+Disease%22%5BTitle%2FAbstract%5D+OR+%222019+Novel+Coronavirus+Infection%22%5BTitle%2FAbstract%5D+OR+%222019-nCoV+Disease%22%5BTitle%2FAbstract%5D+OR+%222019+nCoV+Disease%22%5BTitle%2FAbstract%5D+OR+%222019-nCoV+Diseases%22%5BTitle%2FAbstract%5D+OR+%22Disease%2C+2019-nCoV%22%5BTitle%2FAbstract%5D+OR+COVID19%5BTitle%2FAbstract%5D+OR+%22Coronavirus+Disease+2019%22%5BTitle%2FAbstract%5D+OR+%22Disease+2019%2C+Coronavirus%22%5BTitle%2FAbstract%5D+OR+%22SARS+Coronavirus+2+Infection%22%5BTitle%2FAbstract%5D+OR+%22SARS-CoV-2+Infection%22%5BTitle%2FAbstract%5D+OR+%22Infection%2C+SARS-CoV-2%22%5BTitle%2FAbstract%5D+OR+%22SARS+CoV+2+Infection%22%5BTitle%2FAbstract%5D+OR+%22SARS-CoV-2+Infections%22%5BTitle%2FAbstract%5D+OR+%22COVID-19+Pandemic%22%5BTitle%2FAbstract%5D+OR+%22COVID+19+Pandemic%22%5BTitle%2FAbstract%5D+OR+%22COVID-19+Pandemics%22%5BTitle%2FAbstract%5D+OR+%22Pandemic%2C+COVID-19%22%5BTitle%2FAbstract%5D%29&size=200&ac=no&sort=relevance) | 11:28:55 |
| #17 |  |  | Search: **(Coronavirus Infections[MeSH Terms]) OR ("Coronavirus Infections"[Title/Abstract] OR "Coronavirus Infection"[Title/Abstract] OR "Infection, Coronavirus"[Title/Abstract] OR "Infections, Coronavirus"[Title/Abstract] OR "Middle East Respiratory Syndrome"[Title/Abstract] OR "MERS (Middle East Respiratory Syndrome)"[Title/Abstract])** | [211,687](https://pubmed.ncbi.nlm.nih.gov/?term=%28Coronavirus+Infections%5BMeSH+Terms%5D%29+OR+%28%22Coronavirus+Infections%22%5BTitle%2FAbstract%5D+OR+%22Coronavirus+Infection%22%5BTitle%2FAbstract%5D+OR+%22Infection%2C+Coronavirus%22%5BTitle%2FAbstract%5D+OR+%22Infections%2C+Coronavirus%22%5BTitle%2FAbstract%5D+OR+%22Middle+East+Respiratory+Syndrome%22%5BTitle%2FAbstract%5D+OR+%22MERS+%28Middle+East+Respiratory+Syndrome%29%22%5BTitle%2FAbstract%5D%29&size=200&ac=no&sort=relevance) | 11:28:31 |
| #16 |  |  | Search: **((((Women OR Girls OR Girl OR Woman OR "Women's Groups" OR "Women Groups" OR "Women's Group") OR (Female OR Females)) AND (((Refugees[MeSH Terms]) OR (Refugees[Title/Abstract] OR Refugee[Title/Abstract] OR "Political Asylum Seekers"[Title/Abstract] OR "Asylum Seeker, Political"[Title/Abstract] OR "Asylum Seekers, Political"[Title/Abstract] OR "Political Asylum Seeker"[Title/Abstract] OR "Seekers, Political Asylum"[Title/Abstract] OR "Political Refugees"[Title/Abstract] OR "Political Refugee"[Title/Abstract] OR "Refugee, Political"[Title/Abstract] OR "Refugees, Political"[Title/Abstract] OR "Asylum Seekers"[Title/Abstract] OR "Asylum Seeker"[Title/Abstract] OR "Seeker, Asylum"[Title/Abstract] OR "Seekers, Asylum"[Title/Abstract] OR "Displaced Persons"[Title/Abstract] OR "Displaced Person"[Title/Abstract] OR "Person, Displaced"[Title/Abstract] OR "Persons, Displaced"[Title/Abstract] OR "Internally Displaced Persons"[Title/Abstract] OR "Displaced Person, Internally"[Title/Abstract] OR "Displaced Persons, Internally"[Title/Abstract] OR "Internally Displaced Person"[Title/Abstract])) OR ((Transients and Migrants[MeSH Terms]) OR ("Transients and Migrants"[Title/Abstract] OR "Migrants and Transients"[Title/Abstract] OR Migrants[Title/Abstract] OR Migrant[Title/Abstract])))) AND ((("Health Vulnerability") OR ("Sexual Vulnerability")) OR (Vulnerability))) AND (((Sexual Health[MeSH Terms]) OR ("Sexual Health"[Title/Abstract] OR "Health, Sexual"[Title/Abstract])) OR ((Reproductive Health[MeSH Terms]) OR ("Reproductive Health"[Title/Abstract] OR "Health, Reproductive"[Title/Abstract])))** | [134](https://pubmed.ncbi.nlm.nih.gov/?term=%28%28%28%28Women+OR+Girls+OR+Girl+OR+Woman+OR+%22Women%27s+Groups%22+OR+%22Women+Groups%22+OR+%22Women%27s+Group%22%29+OR+%28Female+OR+Females%29%29+AND+%28%28%28Refugees%5BMeSH+Terms%5D%29+OR+%28Refugees%5BTitle%2FAbstract%5D+OR+Refugee%5BTitle%2FAbstract%5D+OR+%22Political+Asylum+Seekers%22%5BTitle%2FAbstract%5D+OR+%22Asylum+Seeker%2C+Political%22%5BTitle%2FAbstract%5D+OR+%22Asylum+Seekers%2C+Political%22%5BTitle%2FAbstract%5D+OR+%22Political+Asylum+Seeker%22%5BTitle%2FAbstract%5D+OR+%22Seekers%2C+Political+Asylum%22%5BTitle%2FAbstract%5D+OR+%22Political+Refugees%22%5BTitle%2FAbstract%5D+OR+%22Political+Refugee%22%5BTitle%2FAbstract%5D+OR+%22Refugee%2C+Political%22%5BTitle%2FAbstract%5D+OR+%22Refugees%2C+Political%22%5BTitle%2FAbstract%5D+OR+%22Asylum+Seekers%22%5BTitle%2FAbstract%5D+OR+%22Asylum+Seeker%22%5BTitle%2FAbstract%5D+OR+%22Seeker%2C+Asylum%22%5BTitle%2FAbstract%5D+OR+%22Seekers%2C+Asylum%22%5BTitle%2FAbstract%5D+OR+%22Displaced+Persons%22%5BTitle%2FAbstract%5D+OR+%22Displaced+Person%22%5BTitle%2FAbstract%5D+OR+%22Person%2C+Displaced%22%5BTitle%2FAbstract%5D+OR+%22Persons%2C+Displaced%22%5BTitle%2FAbstract%5D+OR+%22Internally+Displaced+Persons%22%5BTitle%2FAbstract%5D+OR+%22Displaced+Person%2C+Internally%22%5BTitle%2FAbstract%5D+OR+%22Displaced+Persons%2C+Internally%22%5BTitle%2FAbstract%5D+OR+%22Internally+Displaced+Person%22%5BTitle%2FAbstract%5D%29%29+OR+%28%28Transients+and+Migrants%5BMeSH+Terms%5D%29+OR+%28%22Transients+and+Migrants%22%5BTitle%2FAbstract%5D+OR+%22Migrants+and+Transients%22%5BTitle%2FAbstract%5D+OR+Migrants%5BTitle%2FAbstract%5D+OR+Migrant%5BTitle%2FAbstract%5D%29%29%29%29+AND+%28%28%28%22Health+Vulnerability%22%29+OR+%28%22Sexual+Vulnerability%22%29%29+OR+%28Vulnerability%29%29%29+AND+%28%28%28Sexual+Health%5BMeSH+Terms%5D%29+OR+%28%22Sexual+Health%22%5BTitle%2FAbstract%5D+OR+%22Health%2C+Sexual%22%5BTitle%2FAbstract%5D%29%29+OR+%28%28Reproductive+Health%5BMeSH+Terms%5D%29+OR+%28%22Reproductive+Health%22%5BTitle%2FAbstract%5D+OR+%22Health%2C+Reproductive%22%5BTitle%2FAbstract%25) | 11:23:03 |
| #15 |  |  | Search: **(((Women OR Girls OR Girl OR Woman OR "Women's Groups" OR "Women Groups" OR "Women's Group") OR (Female OR Females)) AND (((Refugees[MeSH Terms]) OR (Refugees[Title/Abstract] OR Refugee[Title/Abstract] OR "Political Asylum Seekers"[Title/Abstract] OR "Asylum Seeker, Political"[Title/Abstract] OR "Asylum Seekers, Political"[Title/Abstract] OR "Political Asylum Seeker"[Title/Abstract] OR "Seekers, Political Asylum"[Title/Abstract] OR "Political Refugees"[Title/Abstract] OR "Political Refugee"[Title/Abstract] OR "Refugee, Political"[Title/Abstract] OR "Refugees, Political"[Title/Abstract] OR "Asylum Seekers"[Title/Abstract] OR "Asylum Seeker"[Title/Abstract] OR "Seeker, Asylum"[Title/Abstract] OR "Seekers, Asylum"[Title/Abstract] OR "Displaced Persons"[Title/Abstract] OR "Displaced Person"[Title/Abstract] OR "Person, Displaced"[Title/Abstract] OR "Persons, Displaced"[Title/Abstract] OR "Internally Displaced Persons"[Title/Abstract] OR "Displaced Person, Internally"[Title/Abstract] OR "Displaced Persons, Internally"[Title/Abstract] OR "Internally Displaced Person"[Title/Abstract])) OR ((Transients and Migrants[MeSH Terms]) OR ("Transients and Migrants"[Title/Abstract] OR "Migrants and Transients"[Title/Abstract] OR Migrants[Title/Abstract] OR Migrant[Title/Abstract])))) AND ((("Health Vulnerability") OR ("Sexual Vulnerability")) OR (Vulnerability))** | [1,786](https://pubmed.ncbi.nlm.nih.gov/?term=%28%28%28Women+OR+Girls+OR+Girl+OR+Woman+OR+%22Women%27s+Groups%22+OR+%22Women+Groups%22+OR+%22Women%27s+Group%22%29+OR+%28Female+OR+Females%29%29+AND+%28%28%28Refugees%5BMeSH+Terms%5D%29+OR+%28Refugees%5BTitle%2FAbstract%5D+OR+Refugee%5BTitle%2FAbstract%5D+OR+%22Political+Asylum+Seekers%22%5BTitle%2FAbstract%5D+OR+%22Asylum+Seeker%2C+Political%22%5BTitle%2FAbstract%5D+OR+%22Asylum+Seekers%2C+Political%22%5BTitle%2FAbstract%5D+OR+%22Political+Asylum+Seeker%22%5BTitle%2FAbstract%5D+OR+%22Seekers%2C+Political+Asylum%22%5BTitle%2FAbstract%5D+OR+%22Political+Refugees%22%5BTitle%2FAbstract%5D+OR+%22Political+Refugee%22%5BTitle%2FAbstract%5D+OR+%22Refugee%2C+Political%22%5BTitle%2FAbstract%5D+OR+%22Refugees%2C+Political%22%5BTitle%2FAbstract%5D+OR+%22Asylum+Seekers%22%5BTitle%2FAbstract%5D+OR+%22Asylum+Seeker%22%5BTitle%2FAbstract%5D+OR+%22Seeker%2C+Asylum%22%5BTitle%2FAbstract%5D+OR+%22Seekers%2C+Asylum%22%5BTitle%2FAbstract%5D+OR+%22Displaced+Persons%22%5BTitle%2FAbstract%5D+OR+%22Displaced+Person%22%5BTitle%2FAbstract%5D+OR+%22Person%2C+Displaced%22%5BTitle%2FAbstract%5D+OR+%22Persons%2C+Displaced%22%5BTitle%2FAbstract%5D+OR+%22Internally+Displaced+Persons%22%5BTitle%2FAbstract%5D+OR+%22Displaced+Person%2C+Internally%22%5BTitle%2FAbstract%5D+OR+%22Displaced+Persons%2C+Internally%22%5BTitle%2FAbstract%5D+OR+%22Internally+Displaced+Person%22%5BTitle%2FAbstract%5D%29%29+OR+%28%28Transients+and+Migrants%5BMeSH+Terms%5D%29+OR+%28%22Transients+and+Migrants%22%5BTitle%2FAbstract%5D+OR+%22Migrants+and+Transients%22%5BTitle%2FAbstract%5D+OR+Migrants%5BTitle%2FAbstract%5D+OR+Migrant%5BTitle%2FAbstract%5D%29%29%29%29+AND+%28%28%28%22Health+Vulnerability%22%29+OR+%28%22Sexual+Vulnerability%22%29%29+OR+%28Vulnerability%29%29&size=200&ac=no&sort=relevance) | 11:21:29 |
| #14 |  |  | Search: **((Women OR Girls OR Girl OR Woman OR "Women's Groups" OR "Women Groups" OR "Women's Group") OR (Female OR Females)) AND (((Refugees[MeSH Terms]) OR (Refugees[Title/Abstract] OR Refugee[Title/Abstract] OR "Political Asylum Seekers"[Title/Abstract] OR "Asylum Seeker, Political"[Title/Abstract] OR "Asylum Seekers, Political"[Title/Abstract] OR "Political Asylum Seeker"[Title/Abstract] OR "Seekers, Political Asylum"[Title/Abstract] OR "Political Refugees"[Title/Abstract] OR "Political Refugee"[Title/Abstract] OR "Refugee, Political"[Title/Abstract] OR "Refugees, Political"[Title/Abstract] OR "Asylum Seekers"[Title/Abstract] OR "Asylum Seeker"[Title/Abstract] OR "Seeker, Asylum"[Title/Abstract] OR "Seekers, Asylum"[Title/Abstract] OR "Displaced Persons"[Title/Abstract] OR "Displaced Person"[Title/Abstract] OR "Person, Displaced"[Title/Abstract] OR "Persons, Displaced"[Title/Abstract] OR "Internally Displaced Persons"[Title/Abstract] OR "Displaced Person, Internally"[Title/Abstract] OR "Displaced Persons, Internally"[Title/Abstract] OR "Internally Displaced Person"[Title/Abstract])) OR ((Transients and Migrants[MeSH Terms]) OR ("Transients and Migrants"[Title/Abstract] OR "Migrants and Transients"[Title/Abstract] OR Migrants[Title/Abstract] OR Migrant[Title/Abstract])))** | [19,872](https://pubmed.ncbi.nlm.nih.gov/?term=%28%28Women+OR+Girls+OR+Girl+OR+Woman+OR+%22Women%27s+Groups%22+OR+%22Women+Groups%22+OR+%22Women%27s+Group%22%29+OR+%28Female+OR+Females%29%29+AND+%28%28%28Refugees%5BMeSH+Terms%5D%29+OR+%28Refugees%5BTitle%2FAbstract%5D+OR+Refugee%5BTitle%2FAbstract%5D+OR+%22Political+Asylum+Seekers%22%5BTitle%2FAbstract%5D+OR+%22Asylum+Seeker%2C+Political%22%5BTitle%2FAbstract%5D+OR+%22Asylum+Seekers%2C+Political%22%5BTitle%2FAbstract%5D+OR+%22Political+Asylum+Seeker%22%5BTitle%2FAbstract%5D+OR+%22Seekers%2C+Political+Asylum%22%5BTitle%2FAbstract%5D+OR+%22Political+Refugees%22%5BTitle%2FAbstract%5D+OR+%22Political+Refugee%22%5BTitle%2FAbstract%5D+OR+%22Refugee%2C+Political%22%5BTitle%2FAbstract%5D+OR+%22Refugees%2C+Political%22%5BTitle%2FAbstract%5D+OR+%22Asylum+Seekers%22%5BTitle%2FAbstract%5D+OR+%22Asylum+Seeker%22%5BTitle%2FAbstract%5D+OR+%22Seeker%2C+Asylum%22%5BTitle%2FAbstract%5D+OR+%22Seekers%2C+Asylum%22%5BTitle%2FAbstract%5D+OR+%22Displaced+Persons%22%5BTitle%2FAbstract%5D+OR+%22Displaced+Person%22%5BTitle%2FAbstract%5D+OR+%22Person%2C+Displaced%22%5BTitle%2FAbstract%5D+OR+%22Persons%2C+Displaced%22%5BTitle%2FAbstract%5D+OR+%22Internally+Displaced+Persons%22%5BTitle%2FAbstract%5D+OR+%22Displaced+Person%2C+Internally%22%5BTitle%2FAbstract%5D+OR+%22Displaced+Persons%2C+Internally%22%5BTitle%2FAbstract%5D+OR+%22Internally+Displaced+Person%22%5BTitle%2FAbstract%5D%29%29+OR+%28%28Transients+and+Migrants%5BMeSH+Terms%5D%29+OR+%28%22Transients+and+Migrants%22%5BTitle%2FAbstract%5D+OR+%22Migrants+and+Transients%22%5BTitle%2FAbstract%5D+OR+Migrants%5BTitle%2FAbstract%5D+OR+Migrant%5BTitle%2FAbstract%5D%29%29%29&size=200&ac=no&sort=relevance) | 11:20:51 |
| #13 |  |  | Search: **((Refugees[MeSH Terms]) OR (Refugees[Title/Abstract] OR Refugee[Title/Abstract] OR "Political Asylum Seekers"[Title/Abstract] OR "Asylum Seeker, Political"[Title/Abstract] OR "Asylum Seekers, Political"[Title/Abstract] OR "Political Asylum Seeker"[Title/Abstract] OR "Seekers, Political Asylum"[Title/Abstract] OR "Political Refugees"[Title/Abstract] OR "Political Refugee"[Title/Abstract] OR "Refugee, Political"[Title/Abstract] OR "Refugees, Political"[Title/Abstract] OR "Asylum Seekers"[Title/Abstract] OR "Asylum Seeker"[Title/Abstract] OR "Seeker, Asylum"[Title/Abstract] OR "Seekers, Asylum"[Title/Abstract] OR "Displaced Persons"[Title/Abstract] OR "Displaced Person"[Title/Abstract] OR "Person, Displaced"[Title/Abstract] OR "Persons, Displaced"[Title/Abstract] OR "Internally Displaced Persons"[Title/Abstract] OR "Displaced Person, Internally"[Title/Abstract] OR "Displaced Persons, Internally"[Title/Abstract] OR "Internally Displaced Person"[Title/Abstract])) OR ((Transients and Migrants[MeSH Terms]) OR ("Transients and Migrants"[Title/Abstract] OR "Migrants and Transients"[Title/Abstract] OR Migrants[Title/Abstract] OR Migrant[Title/Abstract]))** | [43,511](https://pubmed.ncbi.nlm.nih.gov/?term=%28%28Refugees%5BMeSH+Terms%5D%29+OR+%28Refugees%5BTitle%2FAbstract%5D+OR+Refugee%5BTitle%2FAbstract%5D+OR+%22Political+Asylum+Seekers%22%5BTitle%2FAbstract%5D+OR+%22Asylum+Seeker%2C+Political%22%5BTitle%2FAbstract%5D+OR+%22Asylum+Seekers%2C+Political%22%5BTitle%2FAbstract%5D+OR+%22Political+Asylum+Seeker%22%5BTitle%2FAbstract%5D+OR+%22Seekers%2C+Political+Asylum%22%5BTitle%2FAbstract%5D+OR+%22Political+Refugees%22%5BTitle%2FAbstract%5D+OR+%22Political+Refugee%22%5BTitle%2FAbstract%5D+OR+%22Refugee%2C+Political%22%5BTitle%2FAbstract%5D+OR+%22Refugees%2C+Political%22%5BTitle%2FAbstract%5D+OR+%22Asylum+Seekers%22%5BTitle%2FAbstract%5D+OR+%22Asylum+Seeker%22%5BTitle%2FAbstract%5D+OR+%22Seeker%2C+Asylum%22%5BTitle%2FAbstract%5D+OR+%22Seekers%2C+Asylum%22%5BTitle%2FAbstract%5D+OR+%22Displaced+Persons%22%5BTitle%2FAbstract%5D+OR+%22Displaced+Person%22%5BTitle%2FAbstract%5D+OR+%22Person%2C+Displaced%22%5BTitle%2FAbstract%5D+OR+%22Persons%2C+Displaced%22%5BTitle%2FAbstract%5D+OR+%22Internally+Displaced+Persons%22%5BTitle%2FAbstract%5D+OR+%22Displaced+Person%2C+Internally%22%5BTitle%2FAbstract%5D+OR+%22Displaced+Persons%2C+Internally%22%5BTitle%2FAbstract%5D+OR+%22Internally+Displaced+Person%22%5BTitle%2FAbstract%5D%29%29+OR+%28%28Transients+and+Migrants%5BMeSH+Terms%5D%29+OR+%28%22Transients+and+Migrants%22%5BTitle%2FAbstract%5D+OR+%22Migrants+and+Transients%22%5BTitle%2FAbstract%5D+OR+Migrants%5BTitle%2FAbstract%5D+OR+Migrant%5BTitle%2FAbstract%5D%29%29&size=200&ac=no&sort=relevance) | 11:20:31 |
| #12 |  |  | Search: **(Transients and Migrants[MeSH Terms]) OR ("Transients and Migrants"[Title/Abstract] OR "Migrants and Transients"[Title/Abstract] OR Migrants[Title/Abstract] OR Migrant[Title/Abstract])** | [28,087](https://pubmed.ncbi.nlm.nih.gov/?term=%28Transients+and+Migrants%5BMeSH+Terms%5D%29+OR+%28%22Transients+and+Migrants%22%5BTitle%2FAbstract%5D+OR+%22Migrants+and+Transients%22%5BTitle%2FAbstract%5D+OR+Migrants%5BTitle%2FAbstract%5D+OR+Migrant%5BTitle%2FAbstract%5D%29&size=200&ac=no&sort=relevance) | 11:20:17 |
| #11 |  |  | Search: **(Refugees[MeSH Terms]) OR (Refugees[Title/Abstract] OR Refugee[Title/Abstract] OR "Political Asylum Seekers"[Title/Abstract] OR "Asylum Seeker, Political"[Title/Abstract] OR "Asylum Seekers, Political"[Title/Abstract] OR "Political Asylum Seeker"[Title/Abstract] OR "Seekers, Political Asylum"[Title/Abstract] OR "Political Refugees"[Title/Abstract] OR "Political Refugee"[Title/Abstract] OR "Refugee, Political"[Title/Abstract] OR "Refugees, Political"[Title/Abstract] OR "Asylum Seekers"[Title/Abstract] OR "Asylum Seeker"[Title/Abstract] OR "Seeker, Asylum"[Title/Abstract] OR "Seekers, Asylum"[Title/Abstract] OR "Displaced Persons"[Title/Abstract] OR "Displaced Person"[Title/Abstract] OR "Person, Displaced"[Title/Abstract] OR "Persons, Displaced"[Title/Abstract] OR "Internally Displaced Persons"[Title/Abstract] OR "Displaced Person, Internally"[Title/Abstract] OR "Displaced Persons, Internally"[Title/Abstract] OR "Internally Displaced Person"[Title/Abstract])** | [18,179](https://pubmed.ncbi.nlm.nih.gov/?term=%28Refugees%5BMeSH+Terms%5D%29+OR+%28Refugees%5BTitle%2FAbstract%5D+OR+Refugee%5BTitle%2FAbstract%5D+OR+%22Political+Asylum+Seekers%22%5BTitle%2FAbstract%5D+OR+%22Asylum+Seeker%2C+Political%22%5BTitle%2FAbstract%5D+OR+%22Asylum+Seekers%2C+Political%22%5BTitle%2FAbstract%5D+OR+%22Political+Asylum+Seeker%22%5BTitle%2FAbstract%5D+OR+%22Seekers%2C+Political+Asylum%22%5BTitle%2FAbstract%5D+OR+%22Political+Refugees%22%5BTitle%2FAbstract%5D+OR+%22Political+Refugee%22%5BTitle%2FAbstract%5D+OR+%22Refugee%2C+Political%22%5BTitle%2FAbstract%5D+OR+%22Refugees%2C+Political%22%5BTitle%2FAbstract%5D+OR+%22Asylum+Seekers%22%5BTitle%2FAbstract%5D+OR+%22Asylum+Seeker%22%5BTitle%2FAbstract%5D+OR+%22Seeker%2C+Asylum%22%5BTitle%2FAbstract%5D+OR+%22Seekers%2C+Asylum%22%5BTitle%2FAbstract%5D+OR+%22Displaced+Persons%22%5BTitle%2FAbstract%5D+OR+%22Displaced+Person%22%5BTitle%2FAbstract%5D+OR+%22Person%2C+Displaced%22%5BTitle%2FAbstract%5D+OR+%22Persons%2C+Displaced%22%5BTitle%2FAbstract%5D+OR+%22Internally+Displaced+Persons%22%5BTitle%2FAbstract%5D+OR+%22Displaced+Person%2C+Internally%22%5BTitle%2FAbstract%5D+OR+%22Displaced+Persons%2C+Internally%22%5BTitle%2FAbstract%5D+OR+%22Internally+Displaced+Person%22%5BTitle%2FAbstract%5D%29&size=200&ac=no&sort=relevance) | 11:19:12 |
| #10 |  |  | Search: **((Sexual Health[MeSH Terms]) OR ("Sexual Health"[Title/Abstract] OR "Health, Sexual"[Title/Abstract])) OR ((Reproductive Health[MeSH Terms]) OR ("Reproductive Health"[Title/Abstract] OR "Health, Reproductive"[Title/Abstract]))** | [32,506](https://pubmed.ncbi.nlm.nih.gov/?term=%28%28Sexual+Health%5BMeSH+Terms%5D%29+OR+%28%22Sexual+Health%22%5BTitle%2FAbstract%5D+OR+%22Health%2C+Sexual%22%5BTitle%2FAbstract%5D%29%29+OR+%28%28Reproductive+Health%5BMeSH+Terms%5D%29+OR+%28%22Reproductive+Health%22%5BTitle%2FAbstract%5D+OR+%22Health%2C+Reproductive%22%5BTitle%2FAbstract%5D%29%29&size=200&ac=no&sort=relevance) | 11:18:50 |
| #9 |  |  | Search: **(Reproductive Health[MeSH Terms]) OR ("Reproductive Health"[Title/Abstract] OR "Health, Reproductive"[Title/Abstract])** | [21,062](https://pubmed.ncbi.nlm.nih.gov/?term=%28Reproductive+Health%5BMeSH+Terms%5D%29+OR+%28%22Reproductive+Health%22%5BTitle%2FAbstract%5D+OR+%22Health%2C+Reproductive%22%5BTitle%2FAbstract%5D%29&size=200&ac=no&sort=relevance) | 11:18:36 |
| #8 |  |  | Search: **(Sexual Health[MeSH Terms]) OR ("Sexual Health"[Title/Abstract] OR "Health, Sexual"[Title/Abstract])** | [13,968](https://pubmed.ncbi.nlm.nih.gov/?term=%28Sexual+Health%5BMeSH+Terms%5D%29+OR+%28%22Sexual+Health%22%5BTitle%2FAbstract%5D+OR+%22Health%2C+Sexual%22%5BTitle%2FAbstract%5D%29&size=200&ac=no&sort=relevance) | 11:18:13 |
| #7 |  |  | Search: **(("Health Vulnerability") OR ("Sexual Vulnerability")) OR (Vulnerability)** | [185,328](https://pubmed.ncbi.nlm.nih.gov/?term=%28%28%22Health+Vulnerability%22%29+OR+%28%22Sexual+Vulnerability%22%29%29+OR+%28Vulnerability%29&size=200&ac=no&sort=relevance) | 11:16:36 |
| #6 |  |  | Search: **Vulnerability** | [185,328](https://pubmed.ncbi.nlm.nih.gov/?term=Vulnerability&size=200&ac=no&sort=relevance) | 11:16:16 |
| #5 |  |  | Search: **"Sexual Vulnerability"** | [51](https://pubmed.ncbi.nlm.nih.gov/?term=%22Sexual+Vulnerability%22&size=200&ac=no&sort=relevance) | 11:16:03 |
| #4 |  |  | Search: **"Health Vulnerability"** | [293](https://pubmed.ncbi.nlm.nih.gov/?term=%22Health+Vulnerability%22&size=200&ac=no&sort=relevance) | 11:15:50 |
| #3 |  |  | Search: **(Women OR Girls OR Girl OR Woman OR "Women's Groups" OR "Women Groups" OR "Women's Group") OR (Female OR Females)** | [10,069,294](https://pubmed.ncbi.nlm.nih.gov/?term=%28Women+OR+Girls+OR+Girl+OR+Woman+OR+%22Women%27s+Groups%22+OR+%22Women+Groups%22+OR+%22Women%27s+Group%22%29+OR+%28Female+OR+Females%29&size=200&ac=no&sort=relevance) | 11:14:55 |
| #2 |  |  | Search: **Female OR Females** | [9,731,544](https://pubmed.ncbi.nlm.nih.gov/?term=Female+OR+Females&size=200&ac=no&sort=relevance) | 11:14:18 |
| #1 |  |  | Search: **Women OR Girls OR Girl OR Woman OR "Women's Groups" OR "Women Groups" OR "Women's Group"** | [1,753,515](https://pubmed.ncbi.nlm.nih.gov/?term=Women+OR+Girls+OR+Girl+OR+Woman+OR+%22Women%27s+Groups%22+OR+%22Women+Groups%22+OR+%22Women%27s+Group%22&size=200&ac=no&sort=relevance) | 11:14:08 |

Showing 1 to 26 of 26 entries
